# Supplementary material for: Estimating the global burden of viable Mycobacterium tuberculosis infection: A mathematical modelling study
Source: PLoS Med. 2026 Feb 5;23(2):e1004920. doi: 10.1371/journal.pmed.1004920 (PMC12893653; doi:10.1371/journal.pmed.1004920)
Supplement: S1 File — Additional methodological details, supplementary tables, and figures. (PDF) [file pmed.1004920.s001.pdf]

SUPPLEMENTARY MATERIAL:

Estimating the global burden of viable *Mycobacterium tuberculosis* infection: A mathematical modelling study

Alvaro Schwalb[1,2,3], Peter J. Dodd[4], Hannah M. Rickman[5,6], César A. Ugarte-Gil[7], Katherine C. Horton[1,2], Rein M.G.J. Houben[1,2]

[1] TB Modelling Group, TB Centre, London School of Hygiene and Tropical Medicine, London, United Kingdom; [2] Department of Infectious Disease Epidemiology, London School of Hygiene and Tropical Medicine, London, United Kingdom; [3] Instituto de Medicina Tropical Alexander von Humboldt, Universidad Peruana Cayetano Heredia, Lima, Peru; [4] School of Health and Related Research, University of Sheffield, Sheffield, United Kingdom; [5] Clinical Research Department, London School of Hygiene and Tropical Medicine, London, United Kingdom; [6] Malawi Liverpool Wellcome Programme, Blantyre, Malawi; [7] School of Public and Population Health, University of Texas Medical Branch, Galveston, Texas, United States of America

Correspondence: Alvaro Schwalb, Department of Infectious Disease Epidemiology, London School of Hygiene and Tropical Medicine, Keppel St, London, WC1E 7HT United Kingdom (alvaro.schwalb@lshtm.ac.uk)

GitHub: https://github.com/aschwalbc/MtbInf

Table of Contents:

Supplementary Methods: *4*

Text A. Immunoreactivity surveys 4

Text B. Uncertainty in annual risk of infection estimates 4

Text C. The Stýblo rule 4

Text D. TB prevalence estimates 5

Text E. Immunoreactivity reversion 5

Text F. Self-clearance rates calibration 5

Text G. Model structure 6

Text H. Model formulas 7

Supplementary Tables: *9*

Table A. Immunoreactivity surveys used for annual risk of infection estimates 9

Table B. Comparison of direct and indirect annual risk of infection estimates 15

Table C. Model parameters 16

Table D. Regional relative annual risk of infection as implied by mixing matrices 17

Table E. Adjusted annual risk of infection estimates by age group in 2022 18

Table F. Annual risk of infection estimates in 2014 20

Table G. Number of individuals with viable *Mtb* infection in 2022 22

Table H. Proportion of population with viable *Mtb* infection in 2022 23

Table I. Regional distribution of viable *Mtb* infection in 2022 24

Table J. Top ten countries with the highest number of recent viable *Mtb* infections in 2022 25

Table K. Top ten countries with the highest prevalence of recent viable *Mtb* infections in 2022 26

Table L. Prevalence of viable *Mtb* infection by age and WHO region in 2022 27

Table M. Global viable *Mtb* infection estimates assuming correlated self-clearance rates 31

Table N. Global viable *Mtb* infection estimates under varying self-clearance scenarios 32

Supplementary Figures: *33*

Fig A. Reversion-adjusted annual risk of infection trajectories in the African Region 33

Fig B. Reversion-adjusted annual risk of infection trajectories in the Region of the Americas 34

Fig C. Reversion-adjusted annual risk of infection trajectories in the Eastern-Mediterranean Region 35

Fig D. Reversion-adjusted annual risk of infection trajectories in the European Region 36

Fig E. Reversion-adjusted annual risk of infection trajectories in the South-East Asia Region....................37

Fig F. Reversion-adjusted annual risk of infection trajectories in the Western Pacific Region 38

Fig G. Relative TB incidence per capita in 2022 39

Fig H. Average number of contacts per age group 40

Fig I. Relative annual risk of infection as implied by mixing matrices 41

Fig J. Calibration plots for self-clearance rates under different scenarios 42

References: *44*

Supplementary Methods:

Text A. Immunoreactivity surveys

Direct annual risk of infection (ARI) estimates were obtained from nationally representative immunoreactivity surveys, most of which used the tuberculin skin test (TST) and were implemented among children (**Table A**). The immunoreactivity surveys were conducted in 42 countries over approximately 250 years, resulting in 6 surveys per country-year. These surveys had been previously identified by Cauthen et al. and a systematic search by Houben and Dodd [1,2]. Additionally, we extracted nationally representative surveys from a recent systematic review [3].

Text B. Uncertainty in annual risk of infection estimates

Typically, ARI estimates are reported as point estimates—i.e., without uncertainty. Immunoreactivity surveys report the ARI alongside the sample size and the mean age of participants; this data can be used to conservatively estimate the study's precision, as previously described by Houben and Dodd [2]. Using the information above, the precision can be conservatively estimated as: $\lambda/N\bar{a}$, where N is the sample size, $\bar{a}$ is the mean age of participants, and λ is the force of infection or ARI.

Text C. The Stýblo rule

In 1985, Dr Karel Stýblo formulated a guiding rule for tuberculosis (TB) epidemiology [4]. The Stýblo rule assumes a fixed mathematical relationship, equating an ARI of 1% to an incidence of smear-positive disease of 50 per 100,000 inhabitants and a prevalence of smear-positive disease of 100 per 100,000 inhabitants [4]. This rule implies that one individual with smear-positive TB will cause ten *Mtb* infections per year [4]. However, this widely used rule of thumb was derived from limited observations from the prechemotherapy era, and several studies have since suggested that it may no longer be applicable [5–7]. Transmission from one individual with smear-positive TB might be lower, for reasons such as prompt diagnosis and treatment, or if the individual is a child or has human immunodeficiency virus (HIV) infection, both of which reduce infectiousness, among other factors [6,7]. As seen in the study by Houben and Dodd [2], a revised Stýblo rule was used by fitting a log-normal distribution to data from more recent ARI and TB prevalence estimates [8]: $\beta \sim \text{LogNormal}(\mu = 1.678, \sigma = 0.371)$. Additionally, ARI estimates were adjusted to account for the proportion of prevalent TB that is smear-positive in children and in people living with HIV, using the case detection ratio to compute the mean TB duration by HIV status for each country-year [2].

Text D. TB prevalence estimates

The World Health Organization (WHO) has published a global TB report annually since 1997 [9]. This report includes WHO-generated estimates of TB mortality and incidence, among other metrics. While TB prevalence was previously featured in the document, the latest estimate dates back to 2014. We used the simple approximate relation of prevalence (P), incidence (I), and the average duration of disease ($\overline{D}$) to update TB prevalence estimates [10]. This is expressed as follows: $P = I \times \overline{D}$. We can then express the average duration of disease as the prevalence-to-incidence ratio. We obtained this value by averaging the estimated prevalence and incidence for each country from the last WHO database featuring prevalence estimates (available in the GitHub repository). The country-specific average duration of disease is then applied to current TB incidence estimates (2000 to 2022) to obtain TB prevalence estimates. This method draws from TB incidence estimates that are continually revised and, in some cases, incorporates findings from national TB prevalence surveys.

Text E. Immunoreactivity reversion

We used estimates from previous work that quantified how immunoreactivity reversion may lead to downward bias in ARI estimates derived from cross-sectional surveys [11]. Specifically, we applied the median ratio of true to naïve ARI among children aged 8 to 12 years—the most commonly surveyed age group—within the reversion probability range (15.2–31.4%) reported in the study by Grzybowski and Allen, which analysed five consecutive annual TST surveys conducted in Ontario, Canada in 1959 [12]. This yielded an approximate threefold adjustment (2.9) to account for ARI underestimation due to reversion. To account for additional uncertainty introduced by this correction, we increased the uncertainty interval of all reversion-adjusted ARI estimates by 50%.

Text F. Self-clearance rates calibration

We developed a transition matrix model with a simplified structure, similar to the one described below, comprising sequential '*Infected*' compartments that represent increasing durations since infection and account only for self-clearance. The model tracked an infected cohort beginning in the '*Infected – Year 1*' compartment, with self-clearance and progression through infection-year transitions modelled as competing risks. Infection and reinfection were not included. Self-clearance rates were estimated by calibrating to targets based on the proportion of individuals that

self-cleared or recovered (i.e., no longer harbouring viable *Mtb* infection), as described in the natural history of TB model by Horton et al. [13]. In that model, self-clearance from infection was assigned an uninformed uniform prior (U[0.00,6.00] per year), which was constrained through calibration to multiple data: progression following *Mtb* infection; transitions between non-infectious, asymptomatic, and symptomatic disease states; mortality; duration of infectious TB; and prevalence ratios of non-infectious to infectious TB, and asymptomatic to symptomatic TB.

The resulting proportions no longer harbouring viable *Mtb* infection were 80.9% (95% uncertainty interval [UI]: 65.1, 90.6) at year 1, 91.9% (95% UI: 83.3, 96.3) at year 2, and 97.2% (95% UI: 93.9, 98.7) at year 10. We derived median uncertainty on a logit scale from the lower and upper bounds of the proportion self-cleared, using the logit-transformed uncertainty from the raw data used in the study by Horton et al., which allowed us to estimate a standard deviation at each timepoint [13]. This standard deviation was then used to define a logit-normal calibration likelihood. Due to a lack of data beyond 10 years post-infection, we assumed a proportion of 99.0% (95% UI: 97.8, 99.6) at either year 20 (high self-clearance scenario) or year 50 (low self-clearance scenario) post-infection. As we calibrate to the proportion who self-clear or recover, the complement of these calibration targets (i.e., the proportion that does not self-clear or recover) and, thus, our estimates represent individuals who harbour viable *Mtb* bacilli, including those with TB disease [14].

Calibrated targets were modelled as being normally distributed. Self-clearance rates were drawn from exponential distributions with uninformed priors, assumed to be the same for '*Infected – Year 1*' and '*Infected – Year 2*', and then decreasing thereafter. Posterior estimates were calculated using a Markov chain Monte Carlo algorithm in Stan via R [15,16]. For each scenario, we generated 80,000 parameter sets to be randomly sampled in each model run. The calibration fit was plotted for each scenario and is shown in **Fig D.**

Text G. Model structure

We developed a deterministic compartmental model of *Mycobacterium tuberculosis* (*Mtb*) infection. The model captures the proportional population sizes of each country, and the structure is repeated across multiple 5-year age groups, including the 80+ age group.

The model structure explores the dynamics of *Mtb* infection, reinfection, and self-clearance. Individuals in the '*Uninfected*' compartment are exposed to *Mtb,* as determined by the ARI (symbolised as λ) for their corresponding age category. Upon infection, there is progression through a tunnel model of four '*Infected*' compartments, describing the progression over time from infection. Transitions between infection are fixed values: '*Infected – Year 1*' progresses to '*Infected – Year 2*' at rate $\kappa_1 = 1$, '*Infected – Year 2*' progresses to '*Infected – Years 3 to 10*' at $\kappa_2 = 1$, and '*Infected – Years 3 to 10*' progresses to '*Infected – Years 10+*' at rate $\kappa_3 = 1/8$.

All '*Infected*' compartments can experience self-clearance of infection at varying rates γ_η (with $\eta = 1, \ldots, 4$), informed by calibration; self-clearance effectively returns individuals to the '*Uninfected'* compartment. Individuals who have been distally infected (i.e., infected more than two years ago), '*Infected – Years 3 to 10*' and '*Infected – Years 10+*', are at risk of being reinfected at a rate λ accounting for protection from reinfection π, and thus return to '*Infected – Year 1*'. The model was constructed using R version 4.3.2 for statistical computing and graphics [16].

Text H. Model formulas

To describe the model, we have employed the following variable descriptions, which represent:

- S_α: the proportion of the population in the age group α that is '*Uninfected*'.
- I_α^η: the proportion of the population in the age group α that is in the '*Infected*' compartment for the infection year η, where:
    - $\eta = 1$: '*Infected – Year 1*'
    - $\eta = 2$: '*Infected – Year 2*'
    - $\eta = 3$: '*Infected – Years 3 to 10*'
    - $\eta = 4$: '*Infected – Years 10+*'
- α: the seventeen 5-year age groups from 0-5 ($\alpha = 1$) to 80+ ($\alpha = 17$).
- ε: the ageing term between age groups (ε = 1/5).
- fN_α: the fraction of the total population that belongs to the age group α.
- δ_{ij}: the Kronecker delta, which is 1 when $i = j$ and 0 otherwise.
- π^η: the relative risk of reinfection at infection year η, where $\pi^\eta = 0$ for $\eta = 1$ and $\eta = 2$.
- θ_t: the birth rate per capita at the time t.
- $\lambda_{\alpha,t}$: the force of infection at time t in age group α.
- γ_η: the self-clearance rate from the infection year η.

- κ_{η}: the infection year η transition, where $\kappa_4 = 0$.

To describe the model, we have employed the following differential equations:

$$\frac{dS_\alpha}{dt} = \delta_{\alpha 1} \cdot \frac{\theta_t}{fN_{\alpha,t}} \cdot (1 - S_\alpha) - \lambda_{\alpha,t} \cdot S_\alpha + \sum_{\eta=1}^{4} (\gamma_\eta \cdot I_\alpha^\eta) + (1 - \delta_{\alpha 1}) \cdot \varepsilon \cdot (S_{\alpha-1} - S_\alpha) \cdot \frac{fN_{\alpha-1,t}}{fN_{\alpha,t}}$$

$$\frac{dI_\alpha^\eta}{dt} = -I_\alpha^\eta \cdot \left(\delta_{\alpha 1} \cdot \frac{\theta}{fN_{\alpha,t}} + \gamma_\eta + (1 - \delta_{\eta 4}) \cdot \kappa_\eta + \lambda_{\alpha,t} \cdot \pi^\eta \right) + (1 - \delta_{\eta 1}) \cdot \kappa_{\eta-1} \cdot I_\alpha^{\eta-1}$$
$$+\delta_{\eta 1} \cdot \lambda_{\alpha,t} \cdot \left(S_\alpha + \sum_{\eta'=1}^{4} \pi^{\eta'} \cdot I_\alpha^{\eta'} \right) + (1 - \delta_{\alpha 1}) \cdot \varepsilon \cdot \left(I_{\alpha-1}^{\eta'} - I_\alpha^{\eta'} \right) \cdot \frac{fN_{\alpha-1,t}}{fN_{\alpha,t}}$$

The force of infection ($\lambda_{\alpha,t}$) is derived from national annual risk of infection trajectories spanning 1950 to 2022. These trajectories were subdivided into three age groups (under 15 years, 15 to 45 years, and 45 years and older) and adjusted based on relative ARI. Note that when formulating dynamics in terms of age-stratified proportions in a dynamic population, age-specific mortality rates cancel out.

Supplementary Tables:

Table A. Immunoreactivity surveys used for annual risk of infection estimates

Country (ISO-3)	Survey years (Mid-point)	Number tested	Age (Mid-point)	ARI, % (95%CI)	Reference
**Source:** Cauthen et al. *Bull World Health Organ* 2002 [1].					
AFG	1963 (1963.5)	30,938	10.0	3.06	[17]
AFG	1982 (1982.5)	881	7.5	3.53	[18]
ARG	1960–1961 (1961.0)	1,259	7.5	0.53	[19]
ARG	1967–1968 (1968.0)	1,221	7.0	1.31	[20]
ARG	1967–1968 (1968.0)	3,196	7.0	0.57	[20]
ARG	1974–1975 (1975.0)	3,590	7.0	0.23	[20]
ARG	1974–1978 (1976.5)	26,902	6.5	0.56	[21]
ARG	1979–1980 (1980.0)	443	7.0	0.29	[20]
ARG	1979–1980 (1980.0)	2,125	7.0	0.23	[20]
ARG	1983 (1983.5)	325	7.0	0.26	[20]
BDI	1964 (1964.6)	202	17.5	2.66	[22]
BDI	1982–1984 (1983.5)	912	20.5	1.17	[23]
BHR	1969 (1969.5)	897	7.0	0.90	[24]
BHR	1981 (1981.4)	6,151	7.0	0.20	[24]
BRA	1983 (1983.5)	11,880	7.3	0.39	[25]
BRA	1983 (1983.5)	3,507	7.0	0.56	[25]
BWA	1956–1957 (1957.0)	1,450	6.6	5.79	[26]
BWA	1981–1982 (1982.0)	257	6.5	1.30	[27]
CHN	1979 (1979.5)	10,000*	7.5	1.01	[28]
CMR	1964 (1964.3)	326	7.5	1.32	[29]
CMR	1984 (1984.5)	860	8.5	0.64	[30]
DZA	1949–1952 (1951.1)	110,547	8.5	4.30	[31]
DZA	1976 (1976.5)	262	8.5	1.03	[32]
DZA	1980 (1989.9)	1,844	8.5	0.46	[32]

DZA	1981 (1981.5)	1,117	8.5	0.75	[32]
DZA	1980–1984 (1982.8)	7,514	8.5	0.48	[32]
DZA	1985 (1985.5)	2,378	8.5	0.27	[33]
ETH	1977 (1977.5)	185	8.5	3.81	[34]
ETH	1983 (1983.9)	1,251	8.6	1.30	[35]
GMB	1976 (1976.4)	2,397	9.4	1.92	[36]
IDN	1964–1965 (1965.0)	1,633	7.5	1.64	[37]
IDN	1972 (1972.2)	1,371	9.3	4.73	[38]
IDN	1974 (1974.5)	1,070	8.1	1.76	[39]
IDN	1975 (1975.6)	2,425	8.9	3.76	[39]
IDN	1975 (1975.8)	1,429	8.2	4.26	[39]
IDN	1976 (1976.5)	1,124	8.6	2.17	[40]
IDN	1976 (1976.9)	1,655	8.6	3.09	[40]
IDN	1977 (1977.3)	1,199	8.7	3.92	[39]
IDN	1978 (1978.4)	1,659	8.8	3.47	[38]
IDN	1978 (1978.4)	1,125	8.9	1.77	[39]
IDN	1979 (1979.5)	1,122	8.9	0.89	[39]
IDN	1979 (1979.5)	2,197	8.9	2.12	[39]
IDN	1980 (1980.2)	4,839	8.5	3.80	[39]
IDN	1980 (1980.5)	3,573	8.7	4.03	[39]
IDN	1981 (1981.7)	2,501	8.9	1.75	[40]
IDN	1981 (1981.8)	2,181	8.6	1.77	[40]
IDN	1982 (1982.8)	1,577	9.0	3.42	[39]
IDN	1983 (1983.2)	1,894	8.5	1.28	[39]
IDN	1983 (1983.6)	1,549	8.8	2.29	[38]
IDN	1984 (1984.5)	1,406	8.3	0.66	[39]
IDN	1984 (1984.5)	2,938	8.5	1.72	[39]
IDN	1985 (1985.5)	4,001	8.9	3.92	[39]

IDN	1985 (1985.8)	4,840	8.9	3.48	[39]
IDN	1986 (1986.8)	3,839	8.8	1.83	[40]
IDN	1986 (1986.9)	1,986	8.7	3.07	[40]
IND	1960–1961 (1960.9)	3,788	2.5	1.66	[41]
IND	1961–1963 (1962.2)	7,981	2.5	0.84	[42]
IND	1968–1971 (1969.9)	27,520	3.0	1.70	[43]
IND	1972 (1972.8)	679	3.0	1.40	[43]
IND	1974–1975 (1974.8)	3,805	2.5	1.04	[44]
IND	1977–1978 (1977.9)	1,492	2.5	0.97	[45]
IND	1979 (1979.5)	5,203	2.5	0.99	[44]
KOR	1965 (1965.5)	2,377	2.5	4.23	[46]
KOR	1975 (1975.5)	1,871	2.1	2.32	[46]
KOR	1980 (1980.5)	1,310	2.1	2.36	[46]
KOR	1985 (1985.5)	1,420	2.8	1.97	[47]
KWT	1972 (1972.5)	2,258	4.7	0.36	[48]
KWT	1973 (1973.5)	6,363	4.3	0.29	[48]
KWT	1974 (1974.5)	6,722	4.2	0.20	[48]
KWT	1975 (1975.5)	7,665	4.5	0.29	[48]
KWT	1976 (1976.5)	9,018	5.1	0.44	[48]
KWT	1977 (1977.5)	17,444	5.3	0.29	[48]
KWT	1972–1981 (1978.4)	131,846	5.1	0.26	[48]
KWT	1978 (1978.5)	20,843	5.2	0.31	[48]
KWT	1979 (1979.5)	22,674	5.3	0.24	[48]
KWT	1980 (1980.5)	16,149	5.1	0.12	[48]
KWT	1981 (1981.5)	22,710	5.2	0.22	[48]
LBY	1954 (1954.5)	188	7.5	3.68	[49]
LBY	1959 (1959.7)	361	7.5	2.39	[50]
LBY	1976–1977 (1977.0)	1,827	7.5	0.26	[51]

LBY	1976–1977 (1977.0)	361	7.5	0.26	[51]
LSO	1956–1957 (1957.0)	1,101	4.9	2.78	[26]
LSO	1962–1965 (1963.8)	10,216	4.9	2.83	[52]
LSO	1981–1982 (1982.0)	158	5.2	2.03	[27]
MYS	1976–1977 (1977.0)	1,429	5.5	0.37	[53]
PAK	1961–1962 (1962.0)	769	7.5	3.45	[54]
PAK	1974–1978 (1976.5)	2,289	7.5	1.84	[54]
PHL	1981–1983 (1982.5)	2,038	2.1	1.84	[55]
SYR	1960 (1960.8)	387	7.5	0.74	[56]
SYR	1978 (1978.8)	1,845	6.5	0.26	[57]
SYR	1983 (1983.8)	1,586	6.5	0.30	[57]
SYR	1983 (1983.8)	1,182	6.5	0.14	[57]
THA	1954 (1954.9)	1,578	10.5	2.52	[58]
TZA	1977 (1977.1)	383	9.7	0.92	[59]
TZA	1978 (1978.9)	1,329	10.2	1.30	[60]
TZA	1979 (1979.5)	1,817	10.0	2.16	[61]
TZA	1983–1987 (1985.6)	30,982	10.3	1.11	[62]
**Source:** Houben and Dodd *PLoS Med* 2016 [2].					
AFG	2005–2006 (2005.0)	11,413	20.4	0.80 (0.76, 0.84)	[63]
BGD	1964–1966 (1965.0)	21,658	7.5	0.56 (0.48, 0.64)	[64]
BGD	2007–2009 (2008.0)	9,357	7.0	1.50	[65]
BGD	2007–2009 (2008.0)	8,228	12.0	1.70	[65]
BTN	2009 (2009.0)	835	7.0	0.70 (0.50, 0.90)	[66]
CAF	2011 (2011.0)	2,710	10.0	1.90 (1.70, 2.20)	[67]
CAF	2011 (2011.0)	2,710	10.0	0.80 (0.70, 0.90)	[67]
DJI	1994 (1994.0)	1,505	9.0	2.86	[68]
EGY	1995–1997 (1996.0)	14,766	6.7	0.32	[69]
ETH	1987–1990 (1989.0)	23,695	8.0	1.40	[70]

GMB	2011 (2011.0)	13,386	9.0	1.27 (1.09, 1.49)	[71]
GRC	1981–1991 (1986.0)	544,210	21.0	0.89	[72]
IND	2000–2003 (2002.0)	83,746	4.0	1.50 (1.40, 1.60)	[73]
IND	2009–2010 (2010.0)	18,400	4.0	1.00 (0.80, 1.20)	[73]
KEN	1986–1990 (1988.0)	14,984	8.5	0.60	[74]
KEN	1994–1996 (1995.0)	7,556	9.0	1.10 (0.80, 1.40)	[75]
KEN	2004–2007 (2006.0)	12,107	9.6	1.15 (0.84, 1.48)	[76]
KHM	1995 (1995.0)	1,224	8.0	0.75 (0.56, 0.96)	[77]
KHM	2002 (2002.0)	2,273	6.0	2.06 (1.77, 2.40)	[78]
KOR	1990 (1990.0)	1,210	7.5	1.10	[79]
KOR	1995 (1995.0)	857	7.5	0.50	[80]
LAO	1996–1997 (1997.0)	4,035	8.4	1.10	[81]
MDG	1991–1994 (1993.0)	1,544	8.0	1.29 (0.97, 1.59)	[82]
MWI	1994 (1994.0)	2,696	10.2	1.20 (1.00, 1.40)	[83]
NPL	2006 (2006.0)	17,260	9.0	0.86 (0.49, 1.23)	[84]
PHL	1997 (1997.0)	6,492	7.5	2.30	[85]
SOM	2006 (2006.0)	10,364	9.0	2.70 (2.5, 2.9)	[86]
TZA	1983–1987 (1985.0)	34,427	10.4	1.20	[87]
TZA	1988–1992 (1990.0)	29,696	10.9	1.00	[87]
TZA	1993–1998 (1995.0)	20,592	11.3	0.90	[87]
TZA	2000–2003 (2002.0)	10,239	9.5	0.68 (0.55, 0.81)	[88]
VNM	2006–2007 (2006.0)	21,487	10.0	1.70 (1.50, 1.80)	[89]
YEM	2007 (2007.0)	28,499	9.5	0.05 (0.04, 0.07)	[90]
**Source:** Rickman et al. *Lancet Public Health* 2025 [3].					
BEN	1987–1990 (1989.0)	17,390	7.9	0.44	[91]
BEN	1994 (1994.0)	23,476	8.1	0.50	[91]
BWA	1996 (1996.0)	783	2.3	3.10	[92]
HKG	1999–2000 (2000.0)	21,113	8.0	1.68 (1.61, 1.74)	[93]

KOR	2006 (2006.0)	4,018	6.0	1.90	[94]
LKA	2010 (2010.0)	4,318	10.0	0.40 (0.20, 0.70)	[95]
SAU	2010–2013 (2012.0)	1,369	26.3	0.36	[96]

Direct annual risk of infection (ARI) from immunoreactivity surveys per source used to identify. *Estimated value. ISO-3: International Organization for Standardization 3166-1 alpha-3 codes; AFG: Afghanistan; ARG: Argentina; BDI: Burundi; BEN: Benin; BGD: Bangladesh; BHR: Bahrain; BRA: Brazil; BTN: Bhutan; BWA: Botswana; CAF: Central African Republic; CHN: China; CMR: Cameroon; DZA: Algeria; DJI: Djibouti; EGY: Egypt; ETH: Ethiopia; GMB: Gambia; GRC: Greece; HKG: Hong Kong; IDN: Indonesia; IND: India; KEN: Kenya; KHM: Cambodia; KOR: South Korea; KWT: Kuwait; LAO: Laos; LBY: Libya; LKA: Sri Lanka; LSO: Lesotho; MDG: Madagascar; MWI: Malawi; MYS: Malaysia; NPL: Nepal; PAK: Pakistan; PHL: Philippines; SAU: Saudi Arabia; SOM: Somalia; SYR: Syria; THA: Thailand; TZA: Tanzania; VNM: Vietnam; YEM: Yemen.

Table B. Comparison of direct and indirect annual risk of infection estimates

Country (ISO-3)	Year	Annual risk of infection, % (95%CI)	
		Immunoreactivity surveys (Direct estimates)	TB prevalence (Indirect estimates)
AFG	2006	0.80 (0.72, 0.88)	0.87 (0.69, 1.09)
BGD	2008	1.50 (1.48, 1.52)	1.19 (0.96, 1.47)
		1.70 (1.68, 1.72)	
BTN	2009	0.70 (0.40, 1.24)	0.89 (0.72, 1.09)
CAF	2011	1.90 (1.46, 2.47)	1.60 (1.28, 1.99)
		0.80 (0.62, 1.03)	
GMB	2011	1.27 (0.93, 1.74)	0.35 (0.29, 0.44)
HKG	2000	1.68 (1.55, 1.82)	0.37 (0.30, 0.45)
IND	2002	1.50 (1.31, 1.71)	1.38 (1.03, 1.85)
IND	2010	1.00 (0.67, 1.49)	1.19 (0.91, 1.56)
KEN	2006	1.10 (0.64, 1.90)	1.51 (1.08, 2.11)
KHM	2002	2.06 (1.52, 2.80)	2.98 (2.25, 3.94)
KOR	2006	1.90 (1.86, 1.95)	0.35 (0.29, 0.43)
LKA	2010	0.40 (0.11, 1.40)	0.30 (0.24, 0.37)
NPL	2006	0.86 (0.36, 2.03)	1.32 (1.04, 1.68)
SAU	2013	0.36 (0.34, 0.38)	0.05 (0.04, 0.06)
SOM	2006	2.70 (2.33, 3.13)	1.24 (0.99, 1.55)
TZA	2002	0.68 (0.46, 1.00)	1.72 (1.22, 2.44)
VNM	2006	1.70 (1.42, 2.03)	1.19 (0.91, 1.54)
YEM	2007	0.05 (0.03, 0.09)	0.28 (0.23, 0.34)

Comparison of annual risk of infection (ARI) estimates for the same country-year derived from two sources: immunoreactivity surveys (direct estimates) and WHO TB prevalence data (indirect estimates). AFG: Afghanistan; BGD: Bangladesh; BTN: Bhutan; CAF: Central African Republic; GMB: The Gambia; HKG: Hong Kong; IND: India; KEN: Kenya; KHM: Cambodia; KOR: South Korea; LKA: Sri Lanka; NPL: Nepal; SAU: Saudi Arabia; SOM: Somalia; TZA: Tanzania; VNM: Vietnam; YEM: Yemen.

Table C. Model parameters

Parameter	Description	Notes
λ	Annual risk of infection	Obtained from GP regression, varying by country, year, and age group
γ_η	Self-clearance rate	Obtained from MCMC calibration, varying by infection year
κ_η	Infection year transitions	Fixed values (κ_1 = 1; κ_2 = 1; κ_3 = 1/8)
π	Relative risk of reinfection	0.21(95%CI: 0.14, 0.30) [97], sampled from a beta distribution

GP: Gaussian process; MCMC: Markov chain Monte Carlo.

Table D. Regional relative annual risk of infection as implied by mixing matrices

WHO region	Relative annual risk of infection [95% UI]		
	**Under 15 years***	**15 to 45 years**	**45 years and older**
AFR	1.00	3.38 [2.91, 3.65]	2.91 [2.60, 3.10]
AMR	1.00	3.30 [3.10, 3.53]	2.82 [2.65, 3.05]
EMR	1.00	3.16 [2.89, 3.34]	2.62 [2.50, 2.85]
EUR	1.00	3.32 [3.11, 4.42]	2.88 [2.36, 3.35]
SEA	1.00	3.15 [3.01, 3.42]	2.91 [2.61, 3.17]
WPR	1.00	3.21 [2.97, 3.62]	2.95 [2.60, 3.53]
GLOBAL	1.00	3.29 [2.93, 3.89]	2.85 [2.51, 3.28]

Age-specific relative annual risk of infection by WHO region, as implied by TB incidence and contact mixing matrices. *Individuals under 15 years old were used as the reference age group. Values shown as median, with brackets indicating 95% uncertainty intervals (UI). WHO: World Health Organization; AFR: African Region; AMR: Region of the Americas; EMR: Eastern Mediterranean Region; EUR: European Region; SEA: South-East Asia Region; WPR: Western Pacific Region.

Table E. Adjusted annual risk of infection estimates by age group in 2022

Country (ISO-3)	Annual risk of *Mycobacterium tuberculosis* infection (%) [95% UI]		
	Under 15 years	15 to 45 years	45 years and older
AGO	3.6 [1.5, 8.0]	13.0 [5.5, 29.2]	10.8 [4.6, 24.1]
BGD	3.6 [1.6, 8.3]	11.4 [5.0, 26.2]	10.8 [4.8, 24.8]
BRA	0.4 [0.2, 1.0]	1.4 [0.7, 3.3]	1.2 [0.5, 2.7]
CAF	4.4 [1.9, 11.5]	16.0 [6.9, 41.7]	13.6 [5.9, 35.4]
CHN	0.6 [0.3, 1.4]	2.0 [0.9, 4.3]	1.9 [0.9, 4.2]
COD	3.7 [1.5, 8.9]	12.8 [5.3, 30.4]	11.4 [4.7, 27.2]
COG	3.2 [1.3, 7.6]	11.1 [4.6, 26.5]	9.5 [3.9, 22.5]
ETH	0.9 [0.6, 1.7]	3.5 [2.1, 6.2]	2.7 [1.6, 4.8]
GAB	5.4 [2.1, 13.3]	17.1 [6.6, 41.8]	14.8 [5.7, 36.1]
IDN	4.1 [2.1, 7.6]	13.6 [7.1, 25.3]	11.8 [6.2, 22.0]
IND	2.7 [1.3, 5.2]	8.4 [4.1, 16.2]	7.3 [3.6, 13.9]
KEN	1.6 [0.9, 3.1]	5.9 [3.2, 11.1]	4.9 [2.6, 9.1]
LBR	3.5 [1.5, 8.9]	12.0 [6.2, 24.0]	11.2 [5.6, 21.7]
LSO	3.4 [1.8, 6.7]	11.0 [5.8, 21.6]	9.7 [5.2, 19.1]
MMR	4.0 [2.0, 7.8]	12.4 [6.2, 24.0]	11.2 [5.6, 21.7]
MNG	4.9 [2.2, 10.6]	15.6 [7.0, 34.1]	12.6 [5.7, 27.5]
MOZ	2.9 [1.2, 7.0]	10.0 [3.9, 23.9]	8.9 [3.5, 21.2]
NAM	3.2 [1.5, 6.7]	10.4 [5.0, 22.1]	8.7 [4.2, 18.5]
NGA	1.7 [0.7, 4.6]	5.4 [2.1, 14.7]	4.7 [1.9, 12.8]
PAK	2.7 [1.2, 5.9]	7.9 [3.5, 17.3]	7.6 [3.3, 16.6]
PHL	8.3 [3.8, 19.4]	26.7 [12.1, 62.6]	23.6 [10.7, 55.3]
PNG	4.3 [1.9, 10.5]	13.7 [6.1, 33.6]	11.2 [5.0, 27.5]
PRK	5.1 [2.0, 11.8]	17.3 [6.7, 40.2]	14.0 [5.5, 32.7]

SLE	3.8 [1.6, 9.2]	12.3 [5.1, 29.9]	10.7 [4.4, 25.9]
THA	1.7 [0.9, 3.0]	5.3 [3.0, 9.5]	5.2 [3.0, 9.4]
TZA	2.5 [1.4, 4.5]	8.4 [4.8, 15.2]	7.9 [4.6, 14.4]
UGA	1.3 [0.6, 3.2]	4.4 [1.9, 10.6]	4.0 [1.7, 9.8]
VNM	2.2 [1.0, 5.0]	7.3 [3.4, 16.6]	6.9 [3.2, 15.7]
ZAF	3.6 [1.8, 7.1]	12.1 [6.2, 23.8]	9.8 [5.0, 19.3]
ZMB	2.0 [0.9, 4.5]	7.0 [3.1, 15.6]	6.3 [2.8, 13.9]

Reversion-adjusted, age-specific annual risk of infection for WHO's top 30 high TB burden countries. ISO-3: International Organization for Standardization 3166-1 alpha-3 codes. AGO: Angola; BGD: Bangladesh; BRA: Brazil; CAF: Central African Republic; CHN: China; COD: Democratic Republic of the Congo; COG: Congo; ETH: Ethiopia; GAB: Gabon; IDN: Indonesia; IND: India; KEN: Kenya; LBR: Liberia; MMR: Myanmar; MNG: Mongolia; MOZ: Mozambique; NAM: Namibia; NGA: Nigeria; PAK: Pakistan; PHL: Philippines; PNG: Papua New Guinea; PRK: Democratic People's Republic of Korea; SLE: Sierra Leone; THA: Thailand; TZA: United Republic of Tanzania; UGA: Uganda; VNM: Viet Nam; ZAF: South Africa; ZMB: Zambia.

Table F. Annual risk of infection estimates in 2014

Country (ISO-3)	Annual risk of *Mycobacterium tuberculosis* infection (%) [95% UI]			
	**Unadjusted** [2]	**Age-specific and reversion adjusted**		
	**All ages**	**Under 15 years**	**15 to 45 years**	**45 years and older**
AGO	1.4 [0.7, 2.8]	3.7 [2.0, 7.5]	13.5 [7.4, 27.4]	11.1 [6.1, 22.7]
BGD	1.1 [0.6, 2.3]	3.5 [1.8, 6.9]	11.0 [5.8, 21.7]	10.4 [5.4, 20.5]
BRA	0.1 [0.1, 0.3]	0.4 [0.2, 0.8]	1.4 [0.8, 2.5]	1.1 [0.6, 2.0]
CAF	1.1 [0.5, 2.2]	4.4 [2.3, 8.5]	16.1 [8.3, 30.7]	13.6 [7.1, 26.0]
CHN	0.2 [0.1, 0.5]	0.8 [0.4, 1.5]	2.6 [1.4, 4.7]	2.5 [1.4, 4.6]
COD	1.5 [0.7, 3.0]	3.8 [2.1, 7.8]	13.0 [7.1, 26.6]	11.6 [6.3, 23.7]
COG	1.3 [0.7, 2.7]	3.3 [1.7, 6.0]	11.3 [5.9, 20.8]	9.6 [5.0, 17.7]
ETH	0.5 [0.3, 1.0]	1.5 [0.9, 2.4]	5.5 [3.5, 8.8]	4.2 [2.7, 6.8]
GAB	1.6 [0.8, 3.3]	5.8 [3.1, 11.6]	18.1 [9.9, 36.3]	15.7 [8.5, 31.4]
IDN	1.9 [1.0, 3.4]	4.3 [2.2, 8.1]	14.3 [7.5, 26.9]	12.5 [6.5, 23.4]
IND	0.6 [0.3, 1.1]	3.0 [1.6, 5.8]	9.5 [5.0, 18.3]	8.2 [4.3, 15.8]
KEN	0.6 [0.3, 1.3]	2.8 [2.0, 3.9]	10.2 [7.3, 14.2]	8.3 [6.0, 11.7]
LBR	1.4 [0.7, 2.9]	3.5 [1.9, 6.7]	12.2 [6.5, 23.2]	10.7 [5.7, 20.5]
LSO	1.6 [0.8, 3.2]	4.3 [2.6, 7.2]	14.1 [8.5, 23.4]	12.5 [7.5, 20.7]
MMR	1.2 [0.7, 2.3]	4.7 [3.0, 7.4]	14.4 [9.4, 22.7]	13.0 [8.5, 20.5]
MNG	0.6 [0.3, 1.3]	5.0 [2.9, 8.4]	16.0 [9.4, 22.7]	12.9 [7.6, 21.9]
MOZ	1.6 [0.8, 3.2]	2.9 [1.6, 5.4]	10.0 [5.3, 18.4]	8.9 [4.7, 16.4]
NAM	1.9 [0.9, 3.8]	4.8 [2.8, 7.8]	15.6 [9.2, 25.5]	13.1 [7.7, 21.4]
NGA	0.9 [0.5, 1.6]	1.7 [0.8, 3.1]	5.3 [2.7, 10.0]	4.6 [2.3, 8.7]
PAK	0.9 [0.5, 1.7]	3.0 [1.6, 5.7]	8.8 [4.6, 16.8]	8.4 [4.5, 16.2]
PHL	1.2 [0.6, 2.1]	7.6 [4.0, 14.0]	24.5 [13.0, 44.9]	21.7 [11.5, 39.7]
PNG	1.4 [0.7, 3.0]	4.3 [2.2, 8.4]	13.8 [7.1, 26.7]	11.3 [5.9, 21.9]

PRK	1.4 [0.6, 3.3]	5.0 [2.8, 9.3]	16.9 [9.7, 31.6]	13.7 [7.8, 25.7]
SLE	1.3 [0.6, 2.6]	3.9 [2.0, 7.8]	12.6 [6.6, 25.2]	10.9 [5.7, 21.8]
THA	0.7 [0.4, 1.3]	2.0 [1.2, 3.0]	6.2 [3.9, 9.5]	6.1 [3.9, 9.4]
TZA	1.5 [0.7, 3.3]	2.8 [1.7, 4.8]	9.4 [5.6, 16.0]	8.9 [5.3, 15.1]
UGA	0.3 [0.2, 0.6]	1.4 [0.7, 2.6]	4.5 [2.4, 8.5]	4.2 [2.2, 7.8]
VNM	0.6 [0.3, 1.2]	2.7 [1.6, 4.9]	9.0 [5.1, 16.1]	8.5 [4.8, 15.2]
ZAF	1.8 [0.9, 3.7]	6.0 [3.8, 9.6]	19.9 [12.6, 32.2]	16.2 [10.2, 26.1]
ZMB	1.1 [0.6, 2.2]	2.8 [1.6, 4.8]	9.5 [5.4, 16.5]	8.5 [4.8, 14.8]

Comparison of annual risk of infection estimates for WHO's top 30 high TB burden countries in 2014. Unadjusted estimates were extracted from ARI data from Houben and Dodd [2]. ISO-3: International Organization for Standardization 3166-1 alpha-3 codes. AGO: Angola; BGD: Bangladesh; BRA: Brazil; CAF: Central African Republic; CHN: China; COD: Democratic Republic of the Congo; COG: Congo; ETH: Ethiopia; GAB: Gabon; IDN: Indonesia; IND: India; KEN: Kenya; LBR: Liberia; MMR: Myanmar; MNG: Mongolia; MOZ: Mozambique; NAM: Namibia; NGA: Nigeria; PAK: Pakistan; PHL: Philippines; PNG: Papua New Guinea; PRK: Democratic People's Republic of Korea; SLE: Sierra Leone; THA: Thailand; TZA: United Republic of Tanzania; UGA: Uganda; VNM: Viet Nam; ZAF: South Africa; ZMB: Zambia.

Table G. Number of individuals with viable *Mtb* infection in 2022

WHO region	Recent infections (M) [95% UI]	Recent infections in children (M) [95% UI]	All infections (M) [95% UI]	All infections in children (M) [95% UI]
AFR	23.8 [19.2, 30.9]	4.4 [3.4, 5.8]	51.8 [44.5, 60.6]	7.7 [6.4, 9.5]
AMR	3.9 [2.9, 5.5]	0.4 [0.3, 0.5]	11.0 [8.4, 14.7]	0.6 [0.5, 0.8]
EMR	8.9 [5.4, 15.4]	1.5 [0.9, 2.7]	20.9 [14.6, 29.6]	2.8 [1.8, 4.2]
EUR	3.2 [2.4, 4.3]	0.3 [0.2, 0.3]	13.6 [10.8, 17.1]	0.5 [0.4, 0.6]
SEA	65.5 [40.9, 105.6]	6.8 [4.2, 11.1]	150.7 [106.3, 205.3]	12.6 [8.7, 18.1]
WPR	26.0 [16.6, 42.8]	2.5 [1.6, 4.2]	72.9 [49.7, 102.6]	4.5 [3.3, 6.5]
**GLOBAL**	**133.7 [104.2, 174.4]**	**16.1 [12.8, 20.6]**	**322.4 [271.0, 382.8]**	**29.0 [24.5, 34.9]**

Absolute number of individuals globally and by WHO region infected with viable *Mycobacterium tuberculosis* in 2022. Numbers are in millions (M), with brackets indicating 95% uncertainty intervals (UI). Recent infections are defined as those occurring within the past two years. Children are classified as individuals under 15 years of age. All estimates reflect the low self-clearance scenario. WHO: World Health Organization; AFR: African Region; AMR: Region of the Americas; EMR: Eastern Mediterranean Region; EUR: European Region; SEA: South-East Asia Region; WPR: Western Pacific Region.

Table H. Proportion of population with viable *Mtb* infection in 2022

WHO region	Recent infection prevalence (%) [95% UI]	Proportion of recent infections in children (%) [95% UI]	All infection prevalence (%) [95% UI]	Proportion of all infections in children (%) [95% UI]
AFR	2.0 [1.6, 2.6]	18.4 [17.6, 19.2]	4.4 [3.8, 5.2]	14.9 [14.0, 15.9]
AMR	0.4 [0.3, 0.5]	9.3 [8.8, 9.8]	1.1 [0.8, 1.4]	6.0 [4.6, 7.1]
EMR	1.2 [0.7, 2.0]	17.3 [16.6, 17.9]	2.7 [1.9, 3.8]	13.5 [11.8, 14.9]
EUR	0.3 [0.3, 0.5]	7.9 [7.4, 8.6]	1.5 [1.2, 1.8]	3.7 [2.9, 4.6]
SEA	3.2 [2.0, 5.1]	10.4 [10.1, 10.7]	7.3 [5.2, 9.9]	8.4 [7.5, 9.2]
WPR	1.3 [0.9, 2.2]	9.6 [8.2, 11.6]	3.8 [2.6, 5.3]	6.3 [4.7, 8.4]
**GLOBAL**	**1.7 [1.3, 2.2]**	**12.1 [11.4, 12.8]**	**4.1 [3.4, 4.8]**	**9.0 [8.2, 9.8]**

Proportion of population globally and by WHO region infected with viable *Mycobacterium tuberculosis* in 2022. Values are given percentages (%), with brackets indicating 95% uncertainty intervals (UI). Recent infections are defined as those occurring within the past two years. Children are classified as individuals under 15 years of age. All estimates reflect the low self-clearance scenario. WHO: World Health Organization; AFR: African Region; AMR: Region of the Americas; EMR: Eastern Mediterranean Region; EUR: European Region; SEA: South-East Asia Region; WPR: Western Pacific Region.

Table I. Regional distribution of viable *Mtb* infection in 2022

WHO region	Percentage of global burden of recent infections [95% UI]	Percentage of global burden of all infections – high self-clearance scenario [95% UI]	Percentage of global burden of all infections – low self-clearance scenario [95% UI]
AFR	17.9 [12.9, 24.1]	16.8 [13.5, 20.5]	16.0 [13.0, 19.7]
AMR	2.9 [2.0, 4.3]	3.1 [2.4, 4.3]	3.4 [2.5, 4.7]
EMR	6.8 [3.8, 11.8]	6.6 [4.5, 9.7]	6.5 [4.5, 9.3]
EUR	2.4 [1.7, 3.5]	3.5 [2.7, 4.6]	4.2 [3.2, 5.5]
SEA	49.0 [37.2, 62.4]	47.9 [39.4, 56.6]	46.8 [37.2, 55.8]
WPR	19.7 [12.6, 30.5]	21.6 [15.8, 28.9]	22.5 [15.8, 30.8]

Proportion of global viable *Mycobacterium tuberculosis* infection of population by WHO region in 2022. Values are given as percentages (%), with brackets indicating 95% uncertainty intervals (UI). Recent infections are defined as those occurring within the past two years. Estimates of recent infections differ only minimally between the two long-term clearance scenarios; here, we present results from the high self-clearance scenario. Estimates for all infections are provided and disaggregated based on different scenarios depending on assumptions about long-term self-clearance rates. WHO: World Health Organization; AFR: African Region; AMR: Region of the Americas; EMR: Eastern Mediterranean Region; EUR: European Region; SEA: South-East Asia Region; WPR: Western Pacific Region.

Table J. Top ten countries with the highest number of recent viable *Mtb* infections in 2022

Country (ISO-3)	Recent infections (M) [95% UI]	All infections – high self-clearance scenario (M) [95% UI]	All infections – low self-clearance scenario (M) [95% UI]
India (IND)	39.1 [18.0, 73.6]	84.7 [50.3, 131.3]	94.2 [51.7, 145.6]
Indonesia (IDN)	12.0 [5.8, 22.9]	24.7 [15.2, 37.4]	26.6 [16.2, 40.8]
China (CHN)	11.2 [5.0, 25.5]	32.1 [18.8, 54.2]	40.4 [18.8, 70.3]
Philippines (PHL)	8.8 [3.7, 18.8]	15.9 [8.9, 26.7]	16.5 [9.4, 27.1]
Bangladesh (BGD)	6.5 [2.8, 13.5]	13.1 [7.5, 22.2]	13.9 [7.5, 22.9]
Pakistan (PAK)	6.0 [2.4, 12.2]	12.2 [6.6, 20.8]	13.1 [7.2, 21.3]
DR Congo (COD)	3.4 [1.5, 7.2]	6.6 [3.7, 10.9]	6.7 [3.7, 11.6]
Nigeria (NGA)	3.4 [1.4, 7.9]	6.8 [3.9, 12.0]	7.4 [4.0, 13.2]
Viet Nam (VNM)	2.5 [1.1, 5.4]	5.9 [3.3, 9.7]	6.7 [3.8, 10.6]
South Africa (ZAF)	2.3 [1.0, 4.4]	5.1 [2.9, 8.3]	5.5 [3.2, 8.7]

Absolute number of individuals per country infected with viable Mycobacterium tuberculosis in 2022, showing the top ten countries sorted in descending order by the number of recent infections. Numbers are in millions (M), with brackets indicating 95% uncertainty intervals (UI). Recent infections are defined as those occurring within the past two years. Estimates of recent infection differ only minimally between the two long-term clearance scenarios; here, we present results from the high self-clearance scenario. Estimates for all infections are provided and disaggregated based on different scenarios depending on assumptions about long-term self-clearance rates. ISO-3: International Organization for Standardization 3166-1 alpha-3 codes.

Table K. Top ten countries with the highest prevalence of recent viable *Mtb* infections in 2022

Country (ISO-3)	Recent infection prevalence (%) [95% UI]	All infection prevalence – high self-clearance scenario (%) [95% UI]	All infection prevalence – low self-clearance scenario (%) [95% UI]
Philippines (PHL)	7.7 [3.2, 16.4]	13.9 [7.8, 23.3]	14.4 [8.2, 23.7]
DPR Korea (PRK)	5.4 [2.2, 12.0]	11.3 [6.4, 18.8]	12.0 [7.0, 18.9]
Timor-Leste (TLS)	5.3 [2.2, 11.7]	10.3 [5.7, 18.2]	10.7 [6.3, 18.0]
Gabon (GAB)	5.0 [2.0, 10.3]	9.9 [5.2, 16.1]	10.3 [5.8, 17.4]
Cambodia (KHM)	4.6 [2.3, 8.9]	9.6 [6.1, 15.4]	10.3 [6.1, 16.5]
Mongolia (MNG)	4.6 [2.0, 10.2]	9.3 [5.0, 16.0]	9.7 [5.6, 16.2]
Indonesia (IDN)	4.4 [2.1, 8.3]	9.0 [5.5, 13.6]	9.7 [5.9, 14.8]
Central African Republic (CAF)	4.2 [1.8, 9.5]	7.8 [4.1, 13.6]	8.0 [4.4, 14.1]
Myanmar (MMR)	4.1 [1.9, 8.0]	8.8 [5.3, 13.9]	9.5 [5.5, 14.6]
Papua New Guinea (PNG)	4.0 [1.8, 8.9]	8.1 [4.6, 13.5]	8.5 [5.0, 14.5]

Proportion of population by country infected with viable *Mycobacterium tuberculosis* in 2022, showing the top ten countries sorted in descending order by the prevalence of recent infections. Values are given as percentages (%), with brackets indicating 95% uncertainty intervals (UI). Recent infection is defined as occurring within two years. Estimates for all infections are provided and disaggregated based on different scenarios depending on assumptions about long-term self-clearance rates. ISO-3: International Organization for Standardization 3166-1 alpha-3 codes.

Table L. Prevalence of viable *Mtb* infection by age and WHO region in 2022

WHO region	Age group (years)	Recent infection prevalence (%) [95% UI]	All infection prevalence – high self-clearance scenario (%) [95% UI]	All infection prevalence – low self-clearance scenario (%) [95% UI]
AFR	0–5	0.9 [0.7, 1.1]	1.3 [1.0, 1.6]	1.3 [1.0, 1.6]
	5–10	0.9 [0.7, 1.2]	1.6 [1.4, 2.0]	1.7 [1.4, 2.0]
	10–15	0.9 [0.7, 1.2]	1.8 [1.5, 2.2]	1.9 [1.6, 2.3]
	15–20	2.8 [2.2, 3.5]	4.6 [3.9, 5.6]	4.7 [4.0, 5.8]
	20–25	3.0 [2.4, 3.7]	5.5 [4.7, 6.5]	5.7 [4.8, 6.8]
	25–30	3.0 [2.4, 3.7]	6.0 [5.1, 7.0]	6.3 [5.4, 7.4]
	30–35	3.0 [2.4, 3.8]	6.4 [5.5, 7.4]	6.8 [5.8, 7.8]
	35–40	3.0 [2.3, 3.7]	6.5 [5.6, 7.6]	7.1 [6.1, 8.2]
	40–45	2.9 [2.3, 3.7]	6.6 [5.7, 7.7]	7.3 [6.3, 8.4]
	45–50	2.6 [2.0, 3.3]	6.3 [5.5, 7.2]	7.1 [6.2, 8.2]
	50–55	2.6 [2.0, 3.3]	6.4 [5.5, 7.4]	7.3 [6.3, 8.4]
	55–60	2.6 [2.0, 3.3]	6.4 [5.5, 7.4]	7.4 [6.4, 8.5]
	60–65	2.6 [2.0, 3.3]	6.4 [5.5, 7.4]	7.5 [6.4, 8.6]
	65–70	2.6 [2.0, 3.3]	6.4 [5.5, 7.5]	7.6 [6.5, 8.7]
	70–75	2.6 [2.0, 3.3]	6.5 [5.6, 7.6]	7.7 [6.6, 8.9]
	75–80	2.6 [2.0, 3.3]	6.6 [5.6, 7.7]	7.8 [6.7, 9.0]
	80+	2.6 [2.0, 3.4]	6.7 [5.7, 7.7]	8.0 [6.8, 9.2]
AMR	0–5	0.2 [0.1, 0.2]	0.2 [0.2, 0.3]	0.2 [0.2, 0.3]
	5–10	0.2 [0.1, 0.2]	0.3 [0.2, 0.4]	0.3 [0.2, 0.4]
	10–15	0.2 [0.1, 0.2]	0.3 [0.3, 0.4]	0.3 [0.3, 0.4]
	15–20	0.5 [0.4, 0.7]	0.9 [0.7, 1.1]	0.9 [0.7, 1.1]
	20–25	0.5 [0.4, 0.7]	1.0 [0.8, 1.3]	1.1 [0.9, 1.3]
	25–30	0.5 [0.4, 0.7]	1.1 [0.9, 1.3]	1.2 [0.9, 1.4]
	30–35	0.5 [0.4, 0.7]	1.1 [0.9, 1.4]	1.2 [1.0, 1.5]
	35–40	0.5 [0.4, 0.7]	1.2 [0.9, 1.5]	1.3 [1.1, 1.7]
	40–45	0.5 [0.4, 0.7]	1.2 [0.9, 1.5]	1.4 [1.1, 1.8]

	45–50	0.4 [0.3, 0.6]	1.1 [0.8, 1.4]	1.3 [1.0, 1.8]
	50–55	0.4 [0.3, 0.5]	1.0 [0.8, 1.4]	1.3 [1.0, 1.9]
	55–60	0.3 [0.3, 0.5]	1.0 [0.8, 1.5]	1.3 [0.9, 2.0]
	60–65	0.3 [0.2, 0.5]	1.0 [0.7, 1.6]	1.3 [0.9, 2.2]
	65–70	0.3 [0.2, 0.5]	0.9 [0.7, 1.7]	1.4 [0.9, 2.4]
	70–75	0.3 [0.2, 0.4]	0.9 [0.7, 1.8]	1.4 [0.9, 2.6]
	75–80	0.3 [0.2, 0.4]	0.9 [0.7, 2.0]	1.5 [0.9, 3.0]
	80+	0.3 [0.2, 0.4]	0.9 [0.6, 2.7]	1.6 [0.9, 3.9]
EMR	0–5	0.6 [0.3, 1.0]	0.9 [0.6, 1.4]	0.9 [0.6, 1.3]
	5–10	0.6 [0.4, 1.0]	1.1 [0.7, 1.7]	1.1 [0.7, 1.6]
	10–15	0.6 [0.4, 1.1]	1.2 [0.8, 1.8]	1.3 [0.8, 1.9]
	15–20	1.7 [1.0, 2.9]	2.9 [1.9, 4.4]	2.9 [2.0, 4.4]
	20–25	1.7 [1.0, 2.9]	3.3 [2.3, 4.9]	3.4 [2.4, 5.0]
	25–30	1.6 [0.9, 2.7]	3.3 [2.3, 4.8]	3.5 [2.4, 5.0]
	30–35	1.5 [0.9, 2.5]	3.2 [2.2, 4.5]	3.5 [2.4, 4.9]
	35–40	1.3 [0.8, 2.2]	3.1 [2.2, 4.3]	3.4 [2.4, 4.7]
	40–45	1.3 [0.8, 2.1]	3.0 [2.2, 4.2]	3.4 [2.5, 4.7]
	45–50	1.2 [0.7, 2.1]	3.0 [2.2, 4.2]	3.5 [2.5, 4.8]
	50–55	1.2 [0.7, 2.1]	3.1 [2.2, 4.3]	3.7 [2.6, 5.1]
	55–60	1.3 [0.7, 2.2]	3.3 [2.4, 4.7]	4.0 [2.8, 5.5]
	60–65	1.3 [0.8, 2.3]	3.5 [2.5, 4.9]	4.2 [3.0, 5.8]
	65–70	1.3 [0.7, 2.3]	3.5 [2.5, 4.9]	4.4 [3.1, 5.9]
	70–75	1.3 [0.7, 2.3]	3.6 [2.5, 4.9]	4.5 [3.1, 6.1]
	75–80	1.4 [0.8, 2.4]	3.7 [2.6, 5.2]	4.7 [3.3, 6.4]
	80+	1.2 [0.7, 2.2]	3.4 [2.4, 4.8]	4.5 [3.1, 6.1]
EUR	0–5	0.1 [0.1, 0.2]	0.2 [0.2, 0.3]	0.2 [0.2, 0.3]
	5–10	0.2 [0.1, 0.2]	0.3 [0.2, 0.4]	0.3 [0.3, 0.4]
	10–15	0.2 [0.1, 0.2]	0.3 [0.3, 0.4]	0.4 [0.3, 0.4]
	15–20	0.4 [0.3, 0.6]	0.8 [0.6, 1.0]	0.8 [0.7, 1.0]

	20–25	0.4 [0.3, 0.6]	0.9 [0.8, 1.1]	1.0 [0.9, 1.2]
	25–30	0.5 [0.3, 0.6]	1.1 [1.0, 1.4]	1.3 [1.1, 1.6]
	30–35	0.5 [0.4, 0.7]	1.3 [1.1, 1.6]	1.6 [1.2, 1.9]
	35–40	0.5 [0.4, 0.7]	1.4 [1.1, 1.7]	1.7 [1.3, 2.1]
	40–45	0.4 [0.3, 0.6]	1.3 [1.1, 1.7]	1.7 [1.4, 2.2]
	45–50	0.4 [0.3, 0.5]	1.3 [1.0, 1.6]	1.7 [1.4, 2.2]
	50–55	0.3 [0.3, 0.5]	1.2 [1.0, 1.6]	1.8 [1.4, 2.3]
	55–60	0.3 [0.3, 0.5]	1.3 [1.0, 1.7]	1.9 [1.4, 2.5]
	60–65	0.4 [0.3, 0.5]	1.4 [1.1, 1.9]	2.1 [1.6, 2.8]
	65–70	0.3 [0.2, 0.5]	1.4 [1.1, 1.9]	2.2 [1.6, 2.9]
	70–75	0.3 [0.2, 0.4]	1.3 [1.0, 1.9]	2.2 [1.6, 2.9]
	75–80	0.3 [0.2, 0.3]	1.2 [0.9, 1.8]	2.1 [1.5, 2.8]
	80+	0.2 [0.2, 0.3]	1.3 [1.0, 2.3]	2.5 [1.7, 3.6]
SEA	0–5	1.2 [0.8, 2.0]	1.9 [1.3, 2.7]	1.9 [1.3, 2.8]
	5–10	1.3 [0.8, 2.2]	2.4 [1.7, 3.4]	2.5 [1.7, 3.6]
	10–15	1.3 [0.8, 2.2]	2.7 [1.9, 3.8]	2.8 [2.0, 4.1]
	15–20	3.8 [2.4, 6.1]	6.6 [4.6, 9.3]	6.8 [4.6, 9.7]
	20–25	4.0 [2.5, 6.3]	7.6 [5.4, 10.5]	7.9 [5.5, 11.1]
	25–30	4.0 [2.5, 6.3]	8.1 [5.8, 11.1]	8.6 [6.1, 11.9]
	30–35	4.0 [2.5, 6.3]	8.5 [6.1, 11.6]	9.1 [6.4, 12.5]
	35–40	4.0 [2.5, 6.3]	8.7 [6.3, 11.8]	9.5 [6.7, 12.9]
	40–45	4.0 [2.5, 6.3]	8.9 [6.5, 11.9]	9.8 [6.9, 13.3]
	45–50	3.6 [2.3, 5.6]	8.5 [6.1, 11.3]	9.5 [6.6, 12.8]
	50–55	3.5 [2.3, 5.5]	8.5 [6.1, 11.2]	9.6 [6.7, 12.8]
	55–60	3.5 [2.3, 5.5]	8.4 [6.1, 11.1]	9.6 [6.7, 12.9]
	60–65	3.5 [2.3, 5.4]	8.4 [6.1, 11.1]	9.6 [6.8, 12.9]
	65–70	3.5 [2.3, 5.4]	8.4 [6.0, 11.2]	9.7 [6.7, 13.0]
	70–75	3.5 [2.3, 5.4]	8.4 [6.0, 11.2]	9.7 [6.7, 13.0]
	75–80	3.5 [2.3, 5.4]	8.5 [6.1, 11.2]	9.9 [7.0, 13.1]

	80+	3.4 [2.3, 5.3]	8.4 [6.0, 11.1]	9.8 [6.9, 13.2]
WPR	0–5	0.7 [0.5, 1.2]	1.1 [0.8, 1.7]	1.1 [0.8, 1.7]
	5–10	0.7 [0.4, 1.2]	1.2 [0.9, 1.8]	1.3 [0.9, 1.8]
	10–15	0.7 [0.4, 1.2]	1.4 [1.0, 2.0]	1.5 [1.1, 2.0]
	15–20	2.0 [1.3, 3.2]	3.4 [2.4, 4.7]	3.5 [2.6, 4.8]
	20–25	2.0 [1.3, 3.2]	3.8 [2.8, 5.2]	4.0 [3.0, 5.4]
	25–30	1.9 [1.2, 3.0]	3.9 [2.9, 5.3]	4.3 [3.1, 5.7]
	30–35	1.6 [1.0, 2.6]	3.7 [2.8, 5.1]	4.1 [3.0, 5.7]
	35–40	1.6 [1.0, 2.6]	3.8 [2.8, 5.2]	4.3 [3.0, 5.9]
	40–45	1.6 [1.0, 2.6]	3.9 [2.9, 5.4]	4.5 [3.2, 6.3]
	45–50	1.4 [0.9, 2.3]	3.6 [2.6, 5.2]	4.4 [2.8, 6.5]
	50–55	1.3 [0.8, 2.2]	3.5 [2.5, 5.3]	4.4 [2.7, 6.7]
	55–60	1.2 [0.8, 2.1]	3.5 [2.5, 5.4]	4.4 [2.6, 7.0]
	60–65	1.3 [0.9, 2.2]	3.7 [2.7, 5.6]	4.7 [2.9, 7.1]
	65–70	1.2 [0.7, 2.1]	3.5 [2.4, 5.7]	4.6 [2.6, 7.3]
	70–75	1.1 [0.7, 1.9]	3.3 [2.3, 5.4]	4.5 [2.5, 7.1]
	75–80	1.0 [0.7, 1.8]	3.2 [2.2, 5.4]	4.5 [2.5, 7.1]
	80+	0.9 [0.6, 1.6]	3.0 [2.0, 5.3]	4.5 [2.4, 7.0]

Proportion of global viable *Mycobacterium tuberculosis* infection per age group and by WHO region in 2022. Values are given as percentages (%), with brackets indicating 95% uncertainty intervals (UI). Recent infection is defined as occurring within the past two years. Estimates of recent infection differ only minimally between the two long-term clearance scenarios; here, we present results from the high self-clearance scenario. Estimates for all infections are provided and disaggregated based on different scenarios depending on assumptions about long-term self-clearance rates. WHO: World Health Organization; AFR: African Region; AMR: Region of the Americas; EMR: Eastern Mediterranean Region; EUR: European Region; SEA: South-East Asia Region; WPR: Western Pacific Region.

Table M. Global viable *Mtb* infection estimates assuming correlated self-clearance rates

Recent infections (M) [95% UI]	Recent infection prevalence (%) [95% UI]	All infections (M) [95% UI]	All infection prevalence (%) [95% UI]
132.2 [79.5, 202.2]	1.7 [1.0, 2.6]	287.2 [196.1, 393.8]	3.6 [2.5, 5.0]

Absolute number and proportion of the global population infected with viable *Mycobacterium tuberculosis* in 2022. Values are shown in millions (M) and percentages (%), with brackets indicating 95% uncertainty intervals (UI). Recent infections are defined as those occurring within two years. Estimates reflect correlated self-clearance parameters across countries from the high self-clearance scenario.

Table N. Global viable *Mtb* infection estimates under varying self-clearance scenarios

Self-clearance rate variation	Recent infections (M) [95% UI]	Recent infection prevalence (%) [95% UI]	All infections (M) [95% UI]	All infection prevalence (%) [95% UI]	Proportion self-cleared/recovered at Year 1 (%)	Proportion self-cleared/recovered at Year 2 (%)
–75%	245.1 [206.5, 295.1]	3.1 [2.6, 3.7]	1,683.3 [1,575.9, 1,797.3]	21.3 [19.9, 22.7]	35.7	50.7
–50%	199.5 [165.9, 243.5]	2.5 [2.1, 3.1]	796.1 [730.4, 870.8]	10.1 [9.2, 11.0]	58.2	74.1
–25%	160.3 [131.4, 198.3]	2.0 [1.7, 2.5]	444.3 [401.0, 494.5]	5.6 [5.1, 6.3]	72.5	85.6
Reference	131.7 [107.1, 163.5]	1.7 [1.4, 2.1]	283.3 [249.8, 323.4]	3.6 [3.2, 4.1]	81.6	91.5
+25%	110.7 [89.4, 138.9]	1.4 [1.1, 1.8]	199.4 [172.3, 233.5]	2.5 [2.2, 3.0]	87.5	94.7
+50%	95.0 [76.5, 119.6]	1.2 [1.0, 1.5]	150.9 [128.3, 180.3]	1.9 [1.6, 2.3]	91.4	96.6
+75%	83.0 [66.7, 105.0]	1.0 [0.8, 1.3]	120.2 [101.0, 145.5]	1.5 [1.3, 1.8]	94.0	97.7

Absolute number and proportion of the global population infected with viable *Mycobacterium tuberculosis* in 2022. Values are shown in millions (M) and percentages (%), with brackets indicating 95% uncertainty intervals (UI). Recent infections are defined as those occurring within two years. Estimates reflect systematic variation of self-clearance rates relative to the median estimates from the high self-clearance scenario.

Supplementary Figures:

Fig A. Reversion-adjusted annual risk of infection trajectories in the African Region

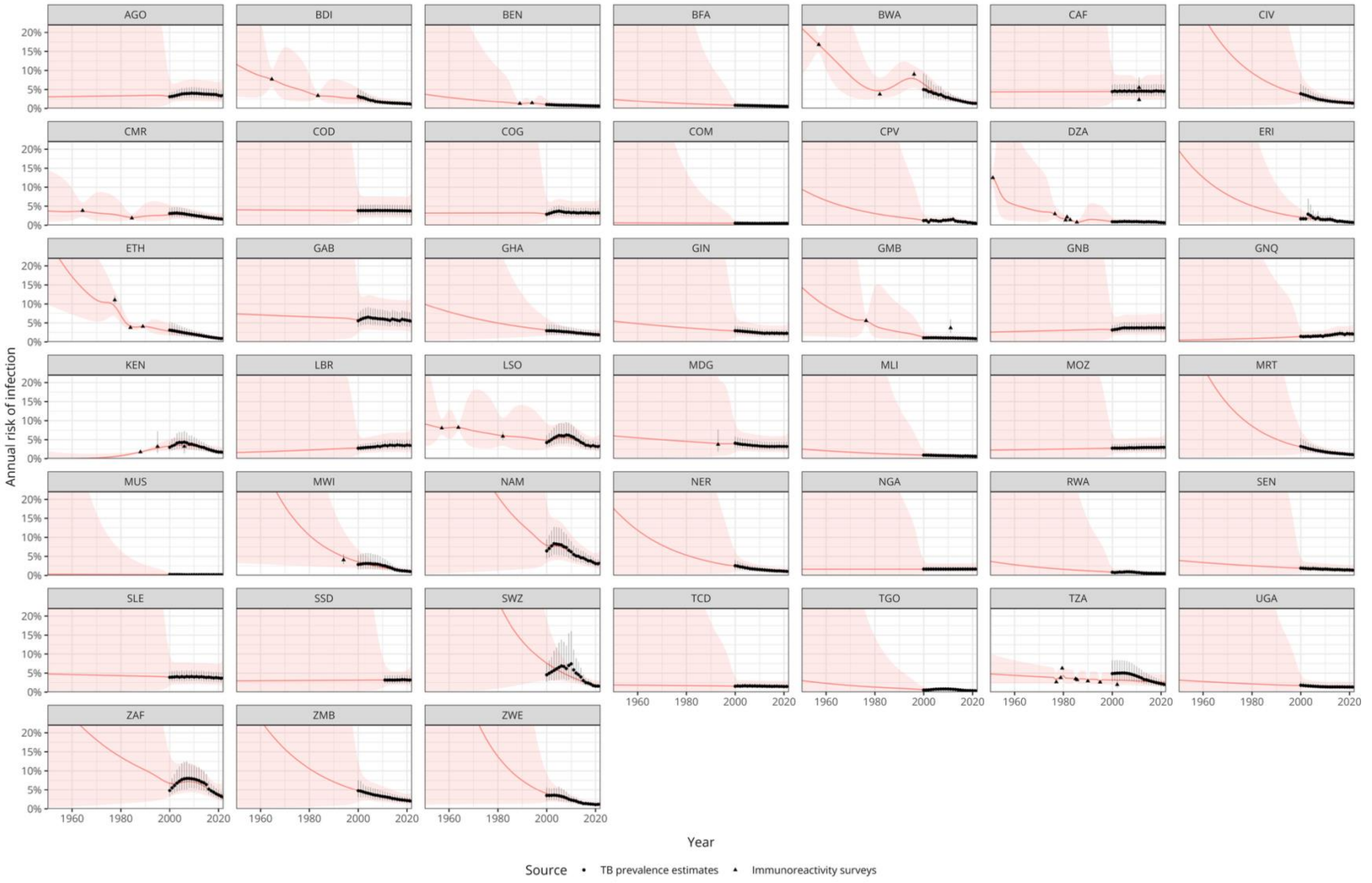

Fig B. Reversion-adjusted annual risk of infection trajectories in the Region of the Americas

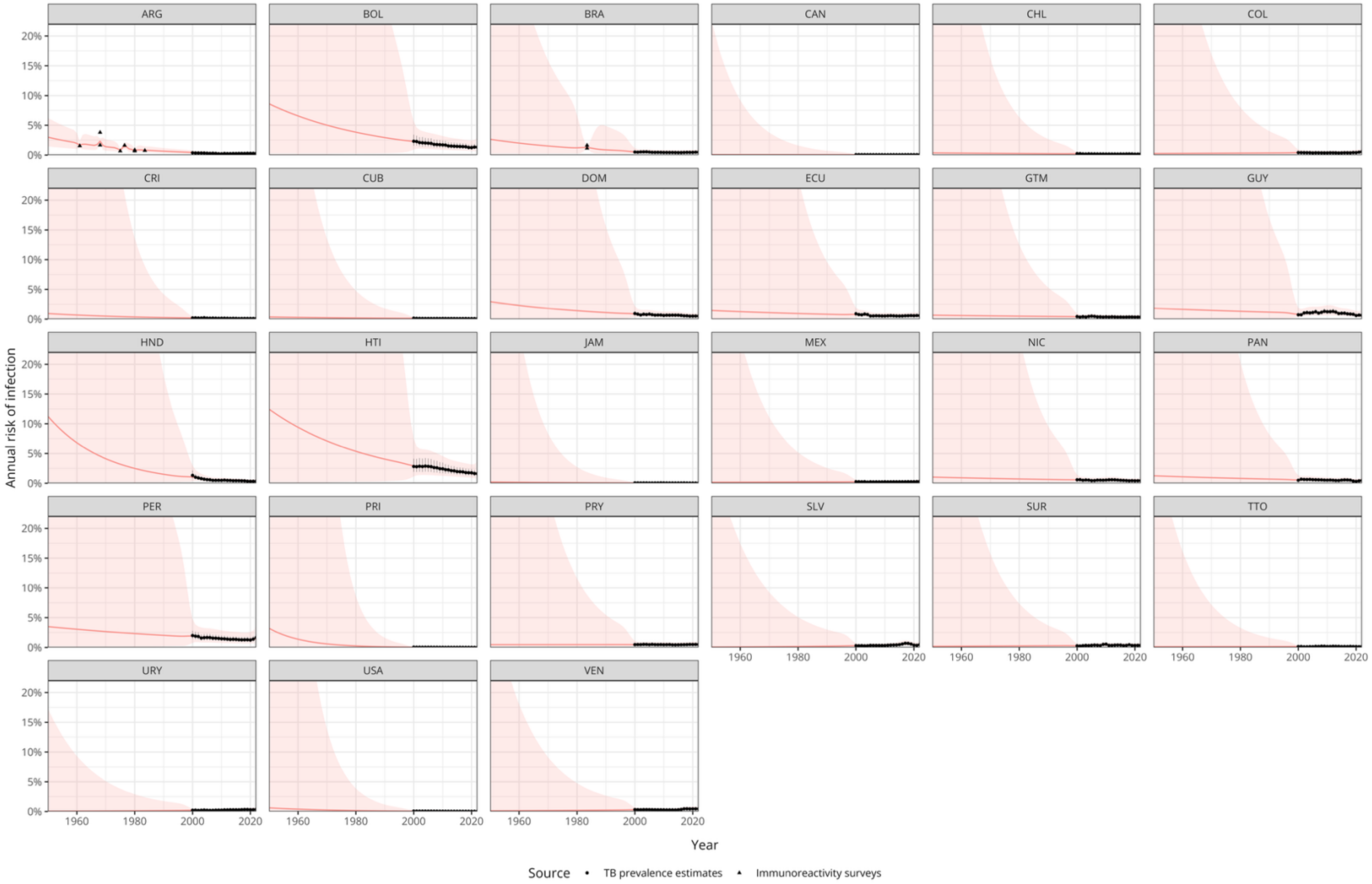

Fig C. Reversion-adjusted annual risk of infection trajectories in the Eastern-Mediterranean Region

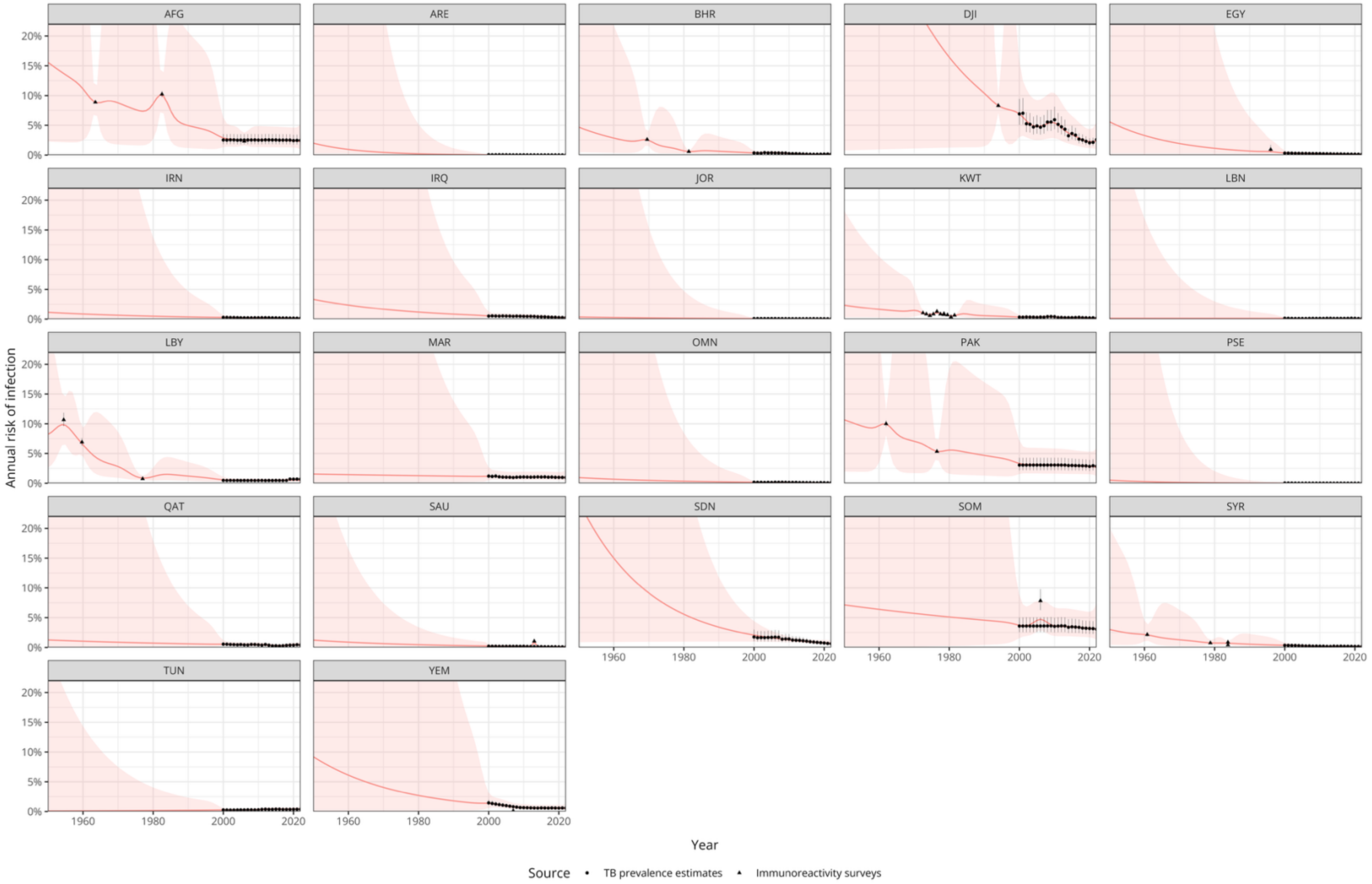

Fig D. Reversion-adjusted annual risk of infection trajectories in the European Region

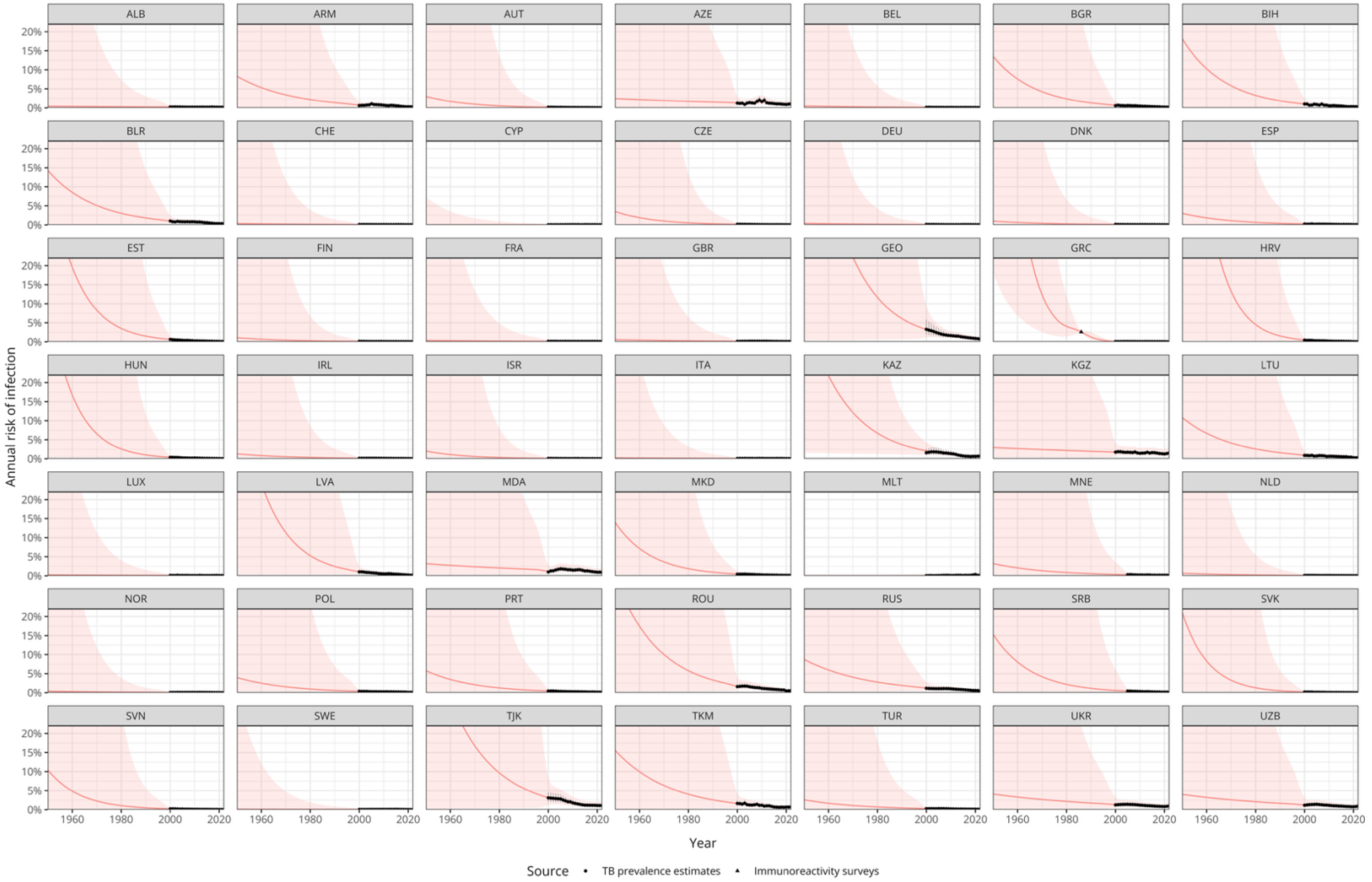

Fig E. Reversion-adjusted annual risk of infection trajectories in the South-East Asia Region

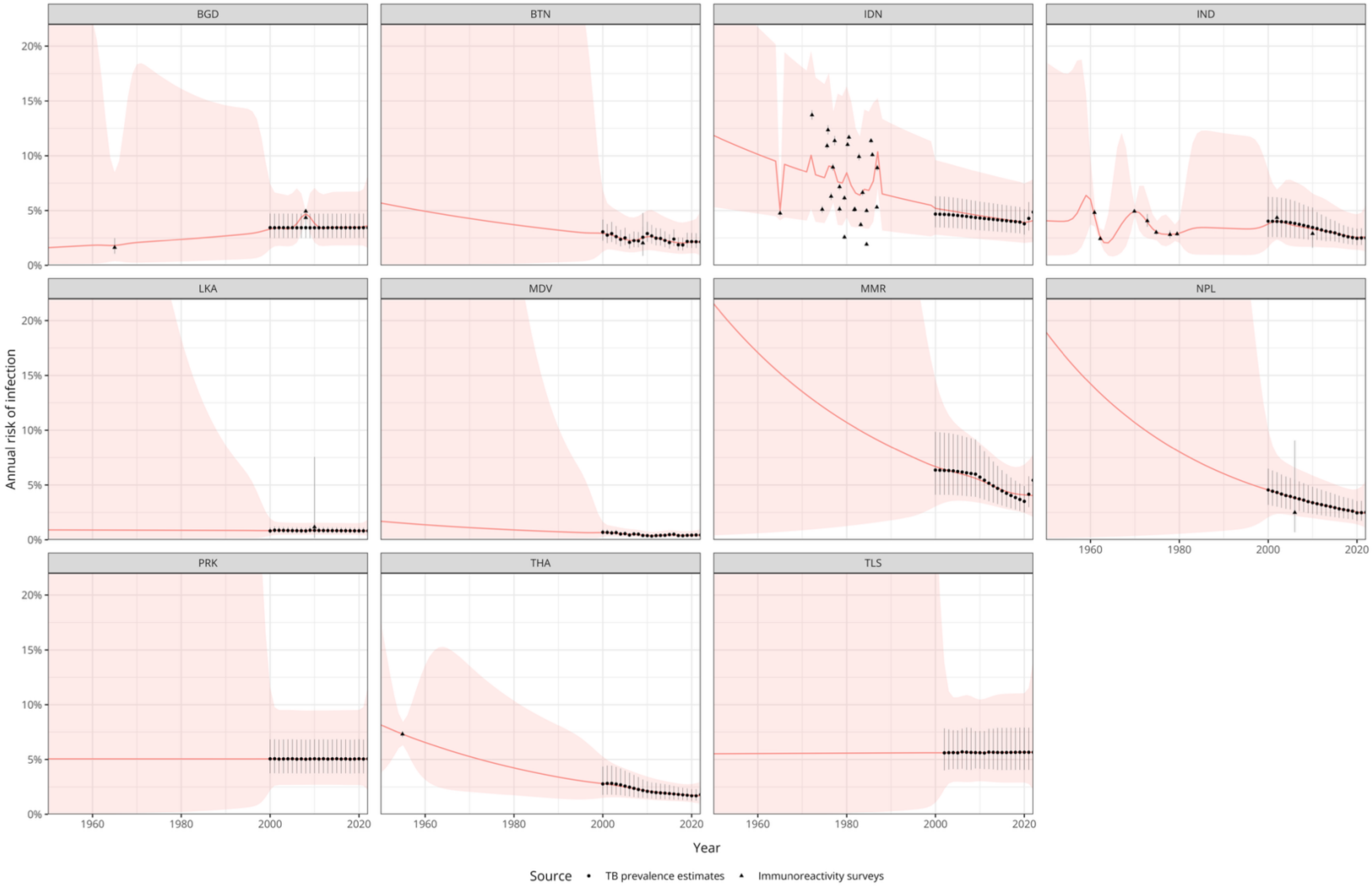

Fig F. Reversion-adjusted annual risk of infection trajectories in the Western Pacific Region

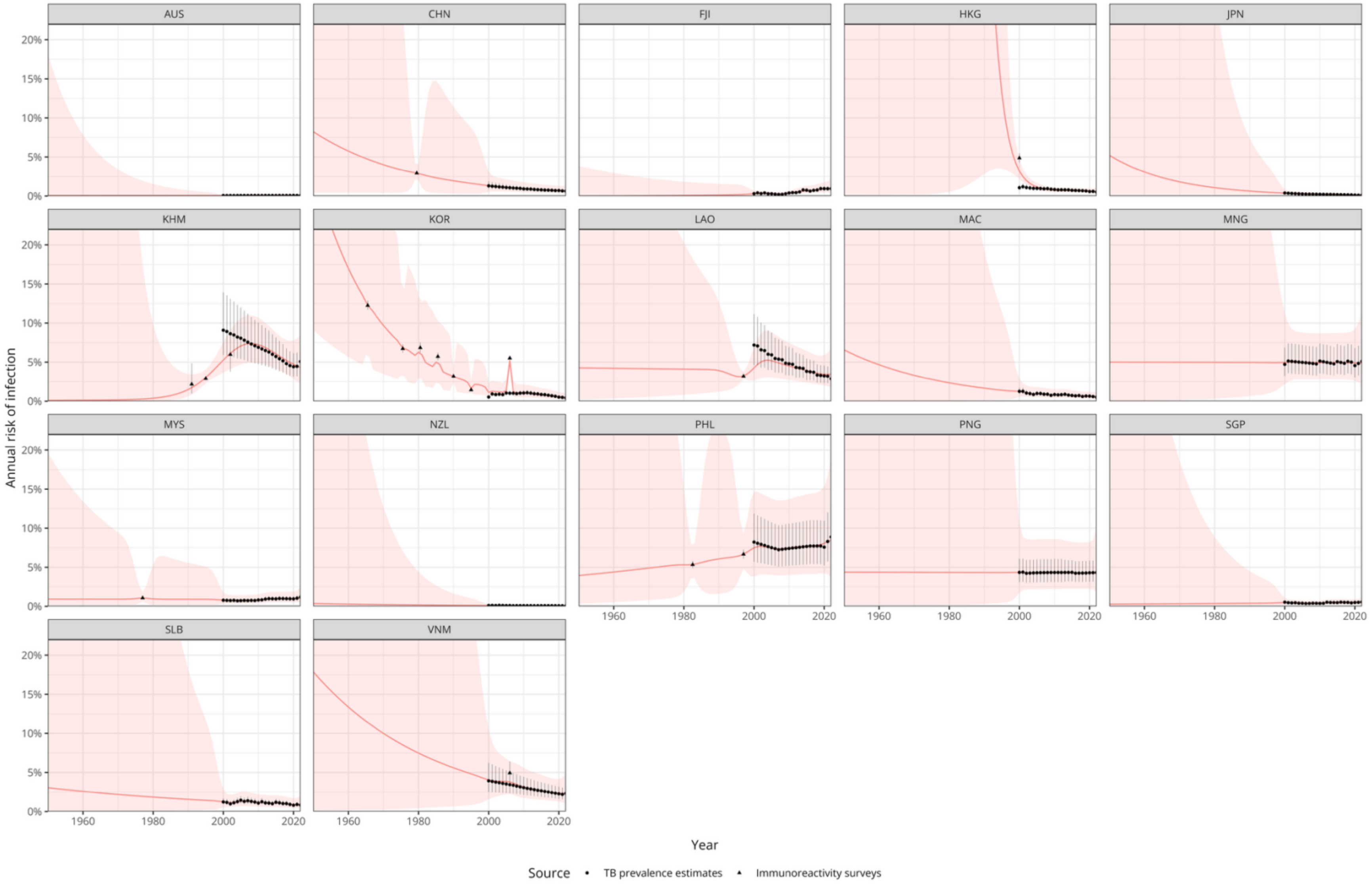

Fig G. Relative TB incidence per capita in 2022

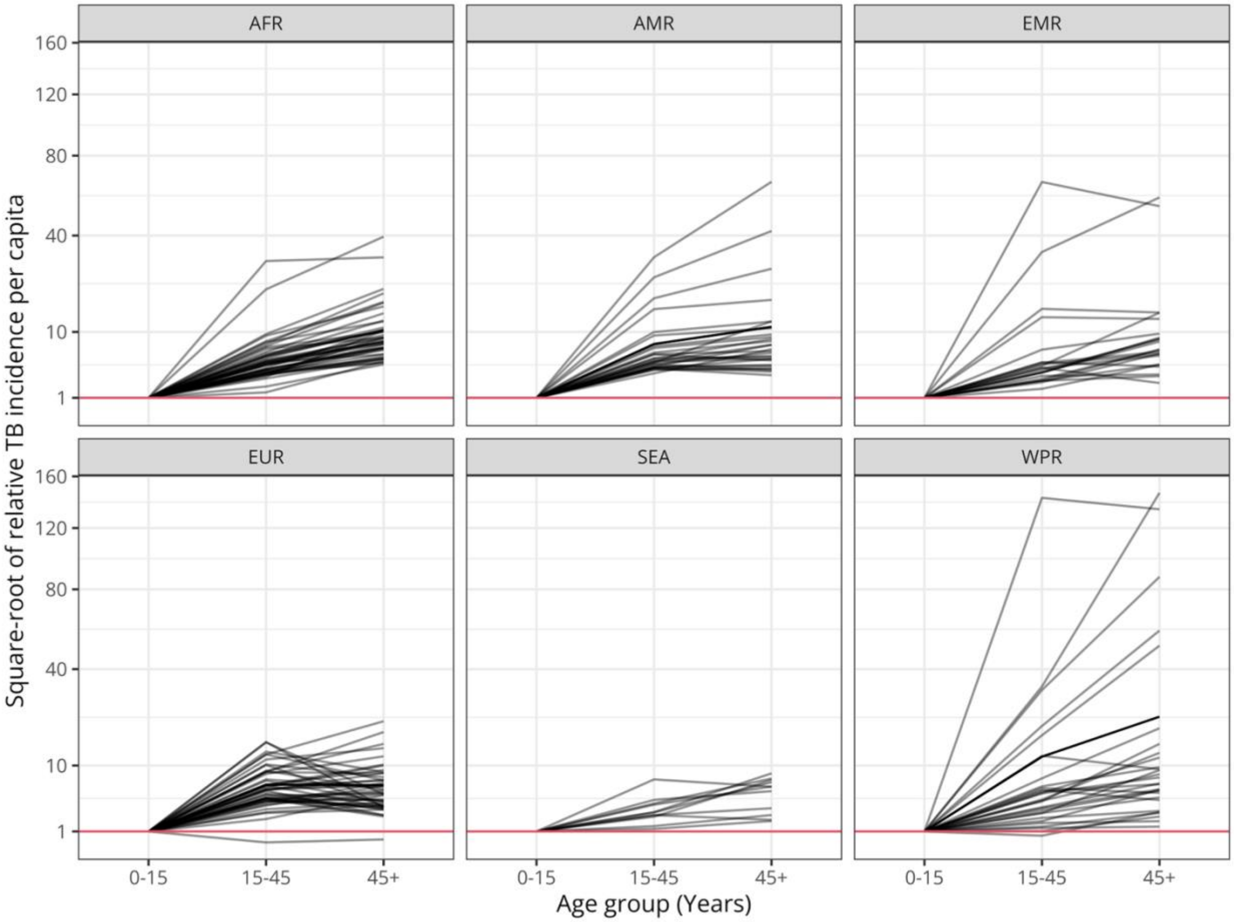

Country-specific relative TB incidence per capita by WHO region in 2022, using children under 15 years of age as the reference group, with the y-axis displayed on a square root scale. Age-specific TB incidence estimates were sourced from WHO [98], and population data from the UN World Population Prospects [99]. WHO: World Health Organization; AFR: African Region; AMR: Region of the Americas; EMR: Eastern Mediterranean Region; EUR: European Region; SEA: South-East Asia Region; WPR: Western Pacific Region; UN: United Nations.

Fig H. Average number of contacts per age group

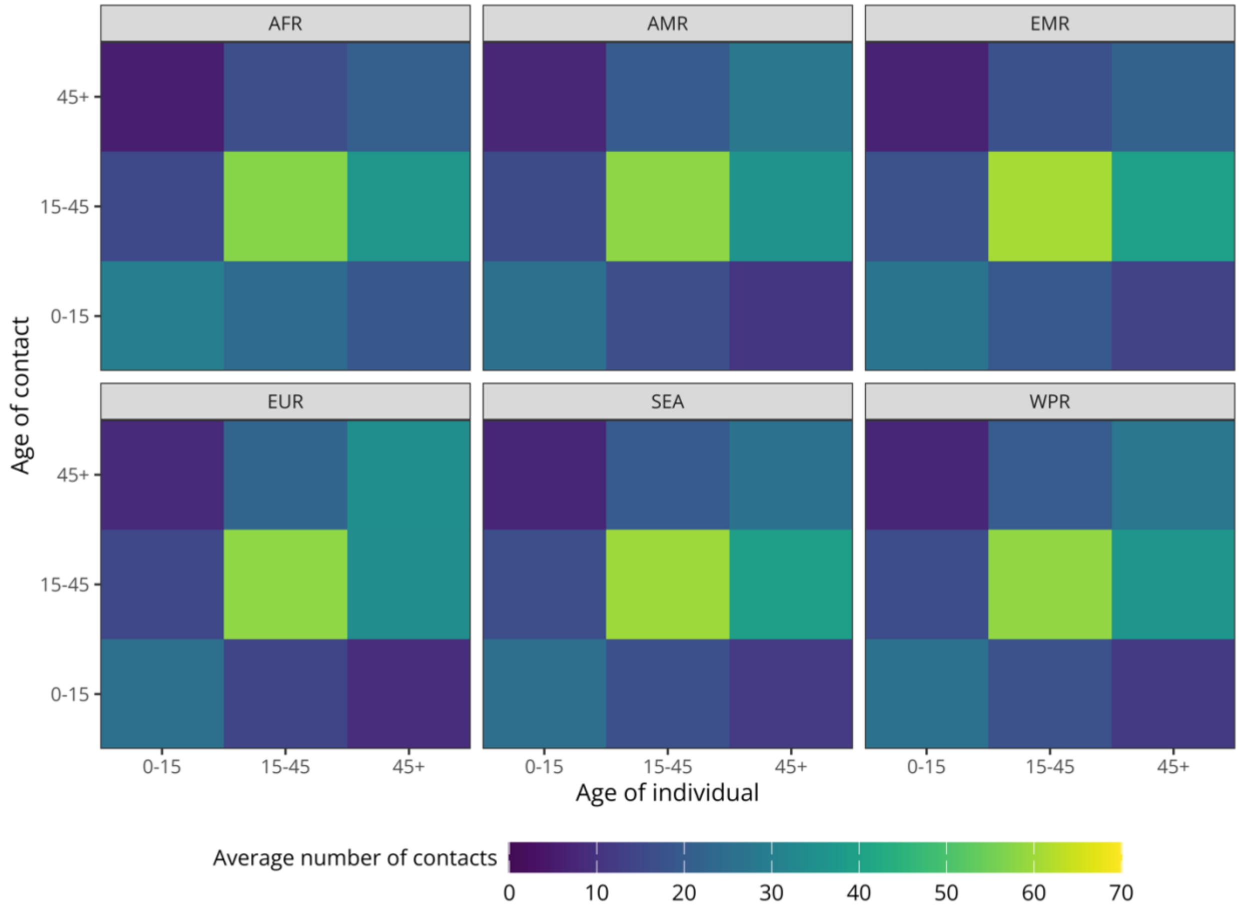

Average number of social contacts per age group by WHO region, based on synthetic country-specific contact mixing matrices developed by Prem et al. [100]. WHO: World Health Organization; AFR: African Region; AMR: Region of the Americas; EMR: Eastern Mediterranean Region; EUR: European Region; SEA: South-East Asia Region; WPR: Western Pacific Region.

Fig I. Relative annual risk of infection as implied by mixing matrices

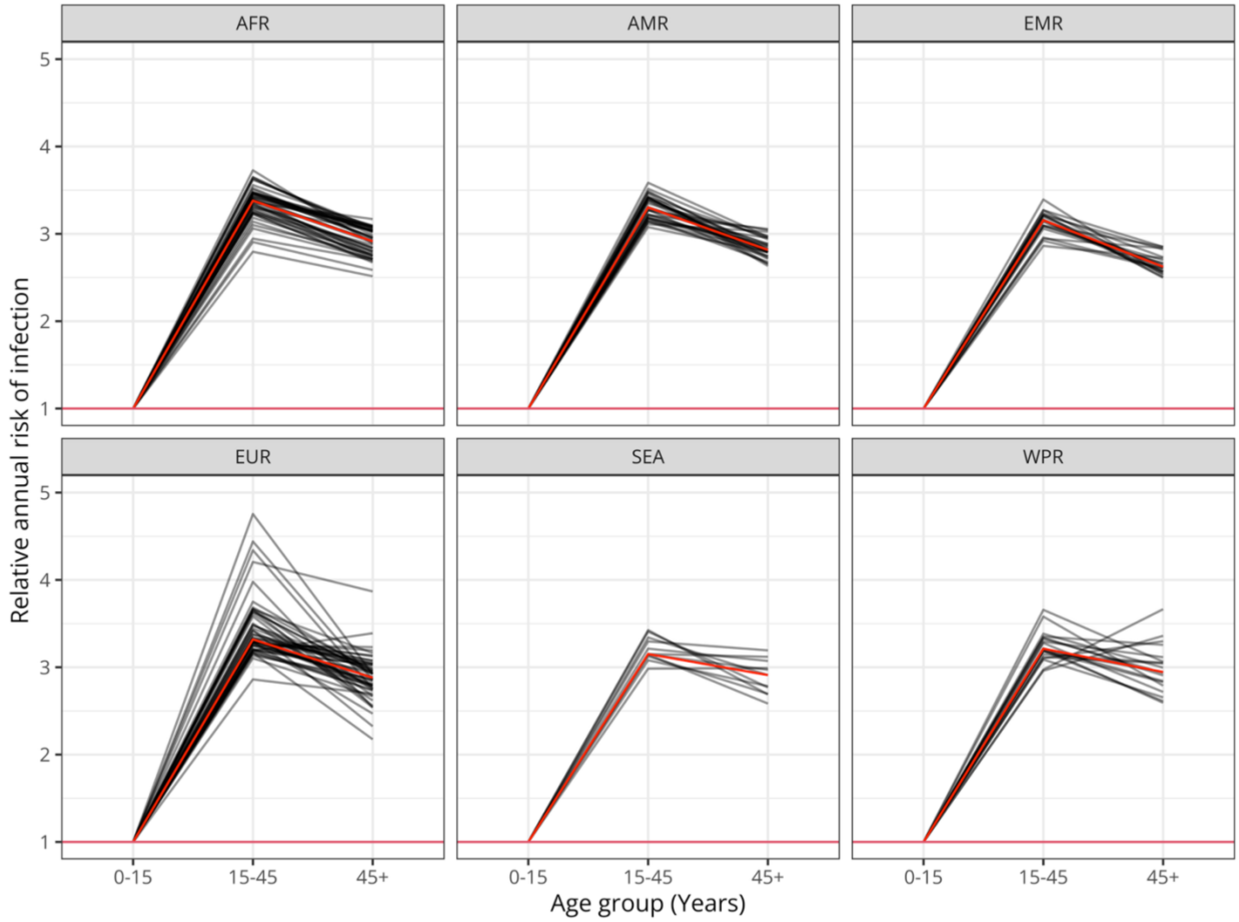

Relative annual risk of *Mycobacterium tuberculosis* infection per country by WHO region in 2022, using children under 15 years of age as the reference group. Black lines show the relative annual risk of infection for individual countries, and red lines indicate the median annual risk by WHO region. WHO: World Health Organization; AFR: African Region; AMR: Region of the Americas; EMR: Eastern Mediterranean Region; EUR: European Region; SEA: South-East Asia Region; WPR: Western Pacific Region.

Fig J. Calibration plots for self-clearance rates under different scenarios

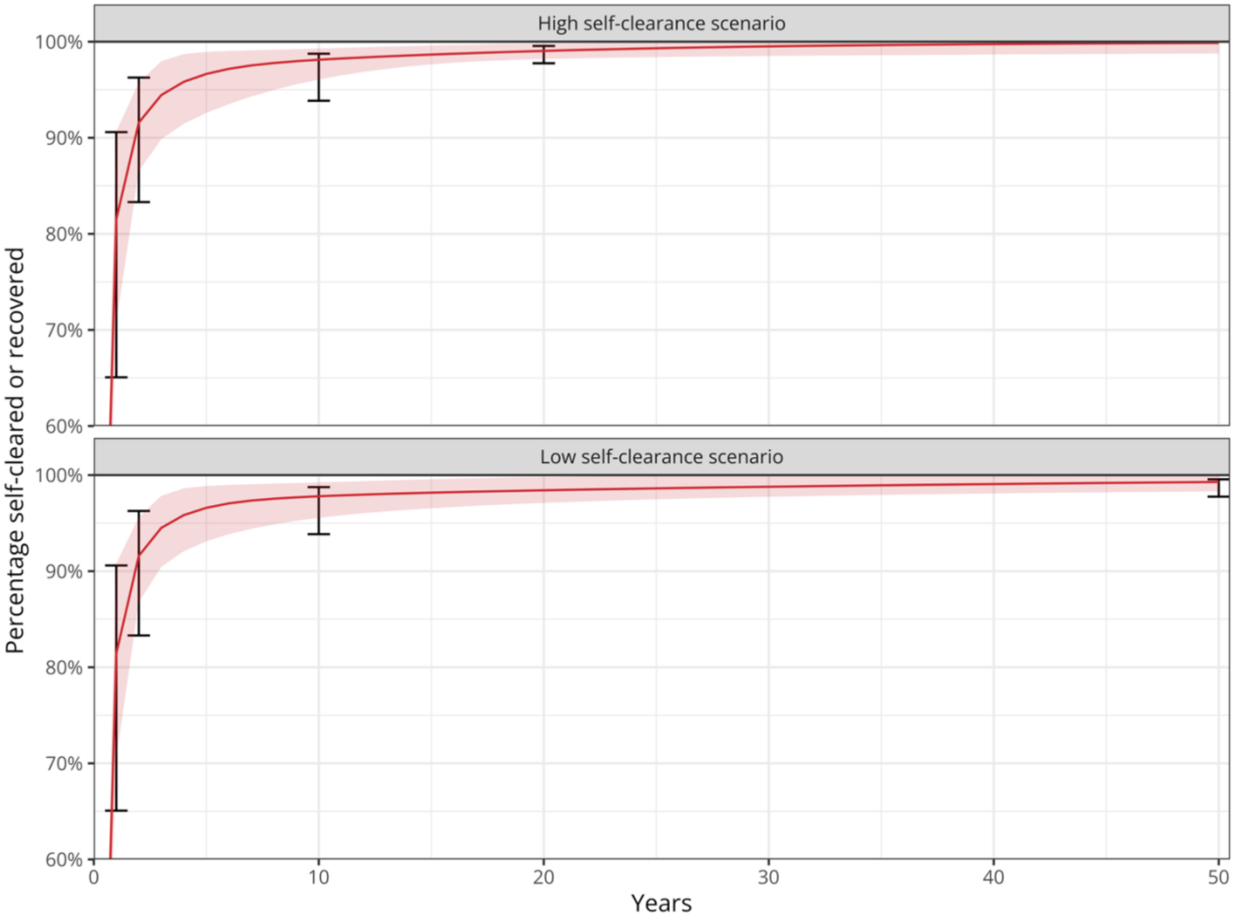

Percentage of an initially infected cohort that has effectively controlled or eliminated *Mycobacterium tuberculosis* infection without developing TB (self-cleared) or after developing TB (recovered)—i.e., no longer harbouring viable infection [13]. Lines represent the median value, and the shaded area shows the lower (2.5% quantile) and upper (97.5% quantile) bounds. Error bars indicate calibration targets at years 1 (65.1, 90.6%), 2 (83.3, 96.3%), and 10 (93.9, 98.7%). Due to limited data beyond year 10, two scenarios were tested, one assuming a range of 97.8 to 99.6% at year 20 (high self-clearance) and another at year 50 (low self-clearance).

Fig K. Model structure

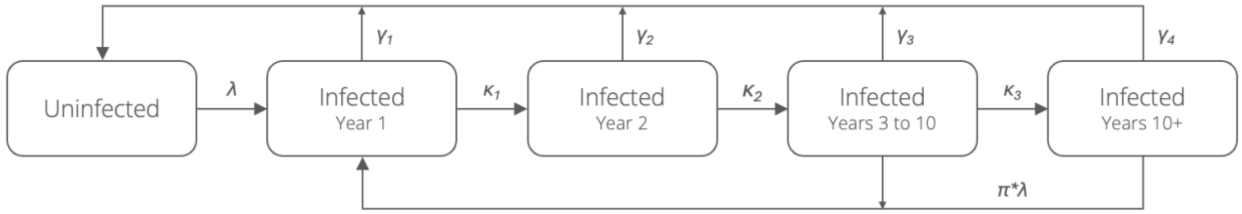

The model structure for a single age group is illustrated. **λ:** Force of infection; **γ:** Self-clearance rates; **κ:** Infection year transitions; **π:** Protection from reinfection. Recent infections are defined as those occurring within two years, i.e., '*Infected – Year 1*' and '*Infected – Year 2*'.

References:

1. Cauthen GM, Pio A, ten Dam HG. Annual risk of tuberculous infection. Bull World Health Organ. 2002;80. Available: https://www.ncbi.nlm.nih.gov/pubmed/12132011

2. Houben RMGJ, Dodd PJ. The Global Burden of Latent Tuberculosis Infection: A Re-estimation Using Mathematical Modelling. PLoS Med. 2016;13: e1002152.

3. Rickman HM, Phiri MD, Feasey HRA, Krutikov M, Shao H, Horton KC, et al. Sex differences in the risk of Mycobacterium tuberculosis infection: a systematic review and meta-analysis of population-based immunoreactivity surveys. Lancet Public Health. 2025;10: e588–e598.

4. Stýblo K. The relationship between the risk of tuberculosis infection and the risk of developing infectious tuberculosis. Bull Int Union Tuberc Lung Dis. 1985;60: 117–119.

5. Dye C. Breaking a law: tuberculosis disobeys Styblo's rule. Bulletin of the World Health Organization. SciELO Public Health; 2008. pp. 4–4. doi:10.2471/BLT07.049510

6. van Leth F, van der Werf MJ, Borgdorff MW. Prevalence of tuberculous infection and incidence of tuberculosis: a re-assessment of the Styblo rule. Bull World Health Organ. 2008;86: 20–26.

7. Bourdin Trunz B, Fine P, Dye C. Effect of BCG vaccination on childhood tuberculous meningitis and miliary tuberculosis worldwide: a meta-analysis and assessment of cost-effectiveness. Lancet. 2006;367: 1173–1180.

8. Dodd PJ, Gardiner E, Coghlan R, Seddon JA. Burden of childhood tuberculosis in 22 high-burden countries: a mathematical modelling study. Lancet Glob Health. 2014;2: e453-9.

9. World Health Organization. Global Tuberculosis Programme - Data. In: WHO [Internet]. 2024 [cited 22 Jul 2024]. Available: https://www.who.int/teams/global-tuberculosis-programme/data

10. Lash TL, VanderWeele TJ, Haneuse S, Rothman KJ. Modern Epidemiology. 4th ed. Philadelphia, PA: Lippincott Williams and Wilkins; 2021.

11. Schwalb A, Emery JC, Dale KD, Horton KC, Ugarte-Gil CA, Houben RMGJ. Impact of Reversion of Mycobacterium tuberculosis Immunoreactivity Tests on the Estimated Annual Risk of Infection. Am J Epidemiol. 2023. doi:10.1093/aje/kwad028

12. Grzybowski S, Allen EA. The challenge of tuberculosis in decline: A study based on the epidemiology of tuberculosis in Ontario, Canada. Am Rev Respir Dis. 1964;90: 707–720.

13. Horton KC, Richards AS, Emery JC, Esmail H, Houben RMGJ. Reevaluating progression and pathways following *Mycobacterium tuberculosis* infection within the spectrum of tuberculosis. Proc Natl Acad Sci U S A. 2023;120: e2221186120.

14. Coussens AK, Zaidi SMA, Allwood BW, Dewan PK, Gray G, Kohli M, et al. Classification of early tuberculosis states to guide research for improved care and prevention: an international Delphi consensus exercise. Lancet Respir Med. 2024. doi:10.1016/S2213-2600(24)00028-6

15. Stan Development Team. Stan Modeling Language Users Guide and Reference Manual, v2.35. 2024. Available: https://mc-stan.org

16. R Core Team. R: A Language and Environment for Statistical Computing. Vienna, Austria: R Foundation for Statistical Computing; 2024. Available: https://www.r-project.org/

17. Khan G. Assignment Report on Tuberculosis Advisory Services (WHO Project: Afghanistan 0033 [UNDP/TA]). Geneva, Switzerland: WHO; 1969.

18. Aneja KS. Assignment Report - National Tuberculosis Survey in Afghanistan. Geneva, Switzerland: WHO; 1983.

19. World Health Organization. Informe sobre la encuesta de prevalencia de la tuberculosis en la Provincia del Chaco. Geneva, Switzerland: WHO; 1961.

20. Balestrino E. Comunicación Oficial. Buenos Aires, Argentina: Instituto Nacional de Epidemiología; 1986.

21. Pan American Health Organization. The tuberculosis situation in Argentina. PAHO Epidemiological Bulletin. 1983;4: 8–10.

22. WHO Regional Office for Africa. A tuberculin sensitivity survey in Burundi. Geneva, Switzerland: WHO; 1964.

23. Service d'Integration de la Lutte Contre la Lèpre et la Tuberculose. Ampleur du problème de la TBC au Burundi calcul de l'incidence réele. Gitega, Burundi: Ministère de la Santé Publique; 1984.

24. Khan MI. Survey of tuberculous infection in schoolchildren in Bahrain. Tubercle. 1982;63: 287–289.

25. Secretaria da Saode e do Meio Ambiente. Estudo da prevaléncia de infecçào tuberculosa na populaçào escolar de 6 - 7 anos no Rio Grande do Sul. Porto Alegre, Brazil: Secretaria da Saode e do Meio Ambiente; 1984.

26. WHO Tuberculosis Research Office. Tuberculosis survey in Basutoland, Bechuanaland, and Swaziland. Geneva, Switzerland: WHO; 1958.

27. Fourie PB, Knoetze K. Prevalence of tuberculosis in Botswana and Lesotho: results of two random sample surveys. S Afr J Epidemiol Infect. 1986;1: 32–37.

28. National Tuberculosis Control and Research Centre. Nation-wide survey for the epidemiology of pulmonary tuberculosis. Zhonghua Jie He He Hu Xi Za Zhi. 1982;5: 67–70.

29. WHO Regional Office for Africa. A tuberculin sensitivity survey in Cameroon. Geneva, Switzerland: WHO; 1964.

30. Delolme HG, Blin P, Roscigno G, Merlin M, Le Mao G, Sentílhes L. Intérêt des enquêtes tuberculíniques par sondage. Yaoundé, Cameroon: OCEAC; 1984.

31. Nyboe J, Soegaard M. Mass BCG vaccination in Algeria, 1949-52, with special reference to statistics on tuberculin testing and BCG vaccination. Geneva, Switzerland: WHO; 1953.

32. Amrane R, Ait-Mesbah H, Chaulet P. Method for evaluating the infection risk (and its trend) in a country with a high prevalence of tuberculosis and where generalised BCG is applied at birth to newly born infants. Bull Int Union Tuberc. 1984;59: 141–143.

33. Amrane R, Ait-Mesbah H, Hani MT. Résultas des mésures de surveillance de la Tuberculose en Algérie de 1980 à 1985. XXVIeme Conférence Mondiale de l' Union Internationale contre la Tuberculose.

34. Fuller GK, Gemeda N, Fuller D, Demerest V. A tuberculin skin test survey in Southwestern Ethiopia. Trop Geogr Med. 1979;31: 365–373.

35. Bleiker MA, Dow N, Ypma H, Meesters H. ITSC 1983/84 Report. The Hague, Netherlands: ITSC; 1984.

36. International Tuberculosis Surveillance Centre. Tuberculin survey in Gambia. The Hague, Netherlands: ITSC; 1976.

37. WHO Regional Office for South-East Asia. Tuberculosis control in Indonesia 1952-65. Geneva, Switzerland: WHO; 1968.

38. International Tuberculosis Surveillance Centre. The risk of tuberculosis infection in the District of Tangerang (Indonesia) derived from the results of tuberculin testing of schoolchildren aged 7 to 10 years, 1972-1983. The Hague, Netherlands: ITSC; 1984.

39. Azuma Y. Tuberculosis Research and Control - Control of bacterial and intestinal diseases. Geneva, Switzerland: WHO; 1982.

40. International Tuberculosis Surveillance Centre. Tuberculin resurveys in two areas in Indonesia. The Hague, Netherlands: ITSC; 1982.

41. Gothi GD, Chakraborty AK, Nair SS, Ganapathy KT, Banerjee GC. Prevalence of tuberculosis in a South Indian District - twelve years after initial survey. Ind J Tub. 1979;26: 122–135.

42. Olakowski T. Assignment report on a tuberculosis longitudinal survey. Geneva, Switzerland: WHO; 1973.

43. Narain R, Krishnamurthy MS, Anantharaman DS. Prevalence of non-specific sensitivity in some parts of India. Indian J Med Res. 1975;63: 1098–1109.

44. Chakraborty AK, Ganapathy KT, Gothi GD. Prevalence of infection among unvaccinated children for tuberculosis surveillance. Indian J Med Res. 1980;72: 7–12.

45. Chakraborty AK, Singh H, Srikantan K, Rangaswamy KR, Krishnamurthy MS, Stephen JA. Tuberculosis in a rural population of South India: Report on five surveys. Indian J Tub. 1982;29: 153–167.

46. Sjögren I. The value of periodical tuberculosis prevalence surveys to assess the epidemiological trend of the problem in developing countries. The Hague, Netherlands: ITSC; 1984.

47. Korean National Tuberculosis Association. Report on the 5th tuberculosis prevalence survey in Korea, 1985. Seoul, South Korea: Ministry of Health and Social Affairs; 1985.

48. Abdel M. Kuwait National Tuberculosis Control Program. Kuwait City, Kuwait: Tuberculosis Control Unit; 1983.

49. WHO Tuberculosis Research Office. Report on BCG assessment work in Libya. Geneva, Switzerland: WHO; 1956.

50. WHO Regional Office for the Eastern Mediterranean. Regional tuberculosis prevalence survey. Geneva, Switzerland: WHO; 1961.

51. Husain SA. Assignment Report: A national tuberculosis prevalence survey in the Socialist People's Libyan Arab Jamahiriyah, February 1976 - December 1977. Geneva, Switzerland: WHO; 1978.

52. Geser A. Report on the initial examination in the WHO-assisted tuberculosis control project Basutoland-2. Geneva, Switzerland: WHO; 1966.

53. Lin HT, Thorup I. An analysis on the first community tuberculin survey in peninsular Malaysia (1976-1977). Kuala Lumpur, Malaysia: National Tuberculosis Programme of Malaysia; 1979.

54. Kaleta J, Nisar M, Chaudry A. Epidemiological situation of tuberculosis in Pakistan - Results of the National Tuberculosis Prevalence Survey 1974-1978. Islamabad, Pakistan: NTP; 1982.

55. National Institute of Tuberculosis. Report on a national tuberculosis prevalence survey in the Republic of the Philippines 1981-1983. Manila, The Philippines: NIT; 1984.

56. WHO Regional Office for the Eastern Mediterranean. Tuberculosis survey in the Syrian Arab Republic. Geneva, Switzerland: WHO; 1962.

57. International Tuberculosis Surveillance Centre. The annual risk of tuberculous infection in the Syrian Arab Republic, 1950-1983. The Hague, Netherlands: ITSC; 1984.

58. WHO Tuberculosis Research Office. Data for the assessment of naturally acquired tuberculin sensitivity in seven countries of Asia. Geneva, Switzerland: WHO; 1955.

59. Stýblo K. Tuberculosis programme implementation: Tanzania. Geneva, Switzerland: WHO; 1986.

60. Broekmans J. The risk of tuberculosis infection in the Dodoma Region, Tanzania. Geneva, Switzerland: WHO; 1978.

61. International Tuberculosis Surveillance Centre. Tuberculin survey in the Shinyanga Region, Tanzania. The Hague, Netherlands: ITSC; 1980.

62. Bleiker MA, Chum HJ, Nkinda SJ, Stýblo K. Tanzania national tuberculin survey. The Hague, Netherlands: ITSC; 1987.

63. Doocy SC, Todd CS, Llainez YB, Ahmadzai A, Burnham GM. Population-based tuberculin skin testing and prevalence of tuberculosis infection in Afghanistan. World Health Popul. 2008;10: 44–53.

64. Begum V, van der Werf MJ, Becx-Bleumink M, Borgdorff MW. Viewpoint: do we have enough data to estimate the current burden of tuberculosis? The example of Bangladesh: Estimating the burden of TB. Trop Med Int Health. 2007;12: 317–322.

65. Hossain S, Zaman K, Banu S, Quaiyum MA, Husain MA, Islam MA, et al. Tuberculin survey in Bangladesh, 2007-2009: prevalence of tuberculous infection and implications for TB control. Int J Tuberc Lung Dis. 2013;17: 1267–1272.

66. Wangchuk LZ, Chadha VK. Annual risk of tuberculous infection among schoolchildren in Bhutan. Int J Tuberc Lung Dis. 2013;17: 468–472.

67. Minime-Lingoupou F, Ouambita-Mabo R, Komangoya-Nzozo A-D, Senekian D, Bate L, Yango F, et al. Current tuberculin reactivity of schoolchildren in the Central African Republic. BMC Public Health. 2015;15: 496.

68. Trébucq A, Guérin N, Ali Ismael H, Bernatas JJ, Sèvre JP, Rieder HL. Prevalence and trends of infection with Mycobacterium tuberculosis in Djibouti, testing an alternative method. Int J Tuberc Lung Dis. 2005;9: 1097–1104.

69. El Ibiary S, de Coster EJ, Tolba FM, van Maaren P, Wasily L, van Cleeff M, et al. Trend in the annual risk of tuberculous infection in Egypt, 1950-1996. Int J Tuberc Lung Dis. 1999;3: 294–299.

70. Azbite M. Tuberculin survey in Ethiopia. Kekkaku. 1992;67: 539–544.

71. Adetifa IMO, Muhammad AK, Jeffries D, Donkor S, Borgdorff MW, Corrah T, et al. A Tuberculin Skin Test Survey and the Annual Risk of Mycobacterium tuberculosis Infection in Gambian School Children. PLoS One. 2015;10: e0139354.

72. Bouros D, Demoiliopoulos I, Moschos M, Panagou P, Demoiliopoulos D, Konstantopoulos S, et al. Tuberculin sensitivity trends in Hellenic army recruits during the period 1981-91. Tuber Lung Dis. 1995;76: 126–129.

73. Chadha VK, Sarin R, Narang P, John KR, Chopra KK, Jitendra R, et al. Trends in the annual risk of tuberculous infection in India. Int J Tuberc Lung Dis. 2013;17: 312–319.

74. Bosman MC, Swai OB, Kwamanga DO, Agwanda R, Idukitta G, Misljenovic O. National tuberculin survey of Kenya, 1986-1990. Int J Tuberc Lung Dis. 1998;2: 272–280.

75. Odhiambo JA, Borgdorff MW, Kiambih FM, Kibuga DK, Kwamanga DO, Ng'ang'a L, et al. Tuberculosis and the HIV epidemic: increasing annual risk of tuberculous infection in Kenya, 1986-1996. Am J Public Health. 1999;89: 1078–1082.

76. Kwamanga D, Chakaya J, Sitienei J, Kalisvaart N, L'herminez R, van der Werf MJ. Tuberculosis transmission in Kenya: results of the third National Tuberculin Survey. Int J Tuberc Lung Dis. 2010;14: 695–700.

77. Norval PY, Roustit C, San KK. From tuberculin to prevalence survey in Cambodia. Int J Tuberc Lung Dis. 2004;8: 299–305.

78. National Tuberculosis Control Program. National TB Prevalence Survey. Cambodia: NTCP; 2005 Aug.

79. Hong YP, Kim SJ, Kwon DW, Chang SC, Lew WJ, Han YC. The sixth Nationwide Tuberculosis Prevalence Survey in Korea, 1990. Tuber Lung Dis. 1993;74: 323–331.

80. Hong YP, Kim SJ, Lew WJ, Lee EK, Han YC. The seventh nationwide tuberculosis prevalence survey in Korea, 1995. Int J Tuberc Lung Dis. 1998;2: 27–36.

81. Arnadottir TH, Soukaseum H, Vangvichit P, Bounmala S, Vos E. Prevalence and annual risk of tuberculosis infection in Laos. Int J Tuberc Lung Dis. 2001;5: 391–399.

82. Champetier de Ribes G, Ranaivoson G, Rakotoherisoa E, Andriamahefazafy B, Blanchy S. [Annual risk of tuberculosis infection in Madagascar: study from 1991 to 1994]. Bull Soc Pathol Exot. 1997;90: 349–352.

83. Salaniponi FML, Kwanjana J, Veen J, Misljenovic O, Borgdorff MW. Risk of infection with Mycobacterium tuberculosis in Malawi: national tuberculin survey 1994. Int J Tuberc Lung Dis. 2004;8: 718–723.

84. Shrestha KB, Malla P, Jha KK, Shakya TM, Akhtar M, Gunneberg C, et al. First national tuberculin survey in Nepal. Int J Tuberc Lung Dis. 2008;12: 909–915.

85. Tupasi TE, Radhakrishna S, Pascual ML, Quelapio MI, Villa ML, Co VM, et al. BCG coverage and the annual risk of tuberculosis infection over a 14-year period in the Philippines assessed from the Nationwide Prevalence Surveys. Int J Tuberc Lung Dis. 2000;4: 216–222.

86. Munim A, Rajab Y, Barker A, Daniel M, Williams B. Risk of Mycobacterium tuberculosis infection in Somalia: national tuberculin survey 2006. East Mediterr Health J. 2008;14: 518–530.

87. Tanzania Tuberculin Survey Collaboration. Tuberculosis control in the era of the HIV epidemic: risk of tuberculosis infection in Tanzania, 1983-1998. Int J Tuberc Lung Dis. 2001;5: 103–112.

88. Egwaga SM, Cobelens FG, Muwinge H, Verhage C, Kalisvaart N, Borgdorff MW. The impact of the HIV epidemic on tuberculosis transmission in Tanzania. AIDS. 2006;20: 915–921.

89. Hoa NB, Cobelens FGJ, Sy DN, Nhung NV, Borgdorff MW, Tiemersma EW. First national tuberculin survey in Viet Nam: characteristics and association with tuberculosis prevalence. Int J Tuberc Lung Dis. 2013;17: 738–744.

90. Al-Absi A, Bassili A, Abdul Bary H, Barker A, Daniels M, Munim A, et al. The decline of tuberculosis in Yemen: evaluation based on two nationwide tuberculin surveys. Int J Tuberc Lung Dis. 2009;13: 1100–1105.

91. Gninafon M, Trébucq A, Rieder HL. Epidemiology of tuberculosis in Benin. Int J Tuberc Lung Dis. 2011;15: 61–66.

92. Lockman S, Tappero JW, Kenyon TA, Rumisha D, Huebner RE, Binkin NJ. Tuberculin reactivity in a pediatric population with high BCG vaccination coverage. Int J Tuberc Lung Dis. 1999;3: 23–30.

93. Leung CC, Yew WW, Tam CM, Chan CK, Chang KC, Law WS, et al. Tuberculin response in BCG vaccinated schoolchildren and the estimation of annual risk of infection in Hong Kong. Thorax. 2005;60: 124–129.

94. Kim HJ, Oh SY, Lee JB, Park YS, Lew WJ. Tuberculin survey to estimate the prevalence of tuberculosis infection of the elementary schoolchildren under high BCG vaccination coverage. Tuberc Respir Dis. 2008;65: 269–276.

95. Wijesinghe PR, Palihawadana P, De Alwis S, Samaraweera S. Annual risk of tuberculosis infection in Sri Lanka: a low prevalent country with a high BCG vaccination coverage in the South-East Asia Region. WHO South East Asia J Public Health. 2013;2: 34–40.

96. Balkhy HH, El Beltagy K, El-Saed A, Aljasir B, Althaqafi A, Alothman AF, et al. Prevalence of Latent Mycobacterium Tuberculosis Infection (LTBI) in Saudi Arabia; Population based survey. Int J Infect Dis. 2017;60: 11–16.

97. Andrews JR, Noubary F, Walensky RP, Cerda R, Losina E, Horsburgh CR. Risk of progression to active tuberculosis following reinfection with Mycobacterium tuberculosis. Clin Infect Dis. 2012;54: 784–791.

98. World Health Organization. Global Tuberculosis Report 2023. Geneva, Switzerland: WHO; 2023. Available: https://iris.who.int/bitstream/handle/10665/373828/9789240083851-eng.pdf?sequence=1

99. United Nations. World Population Prospects - Population Division. In: World Population Prospects 2022 [Internet]. [cited Jun 2023]. Available: https://population.un.org/wpp/

100. Prem K, van Zandvoort K, Klepac P, Eggo RM, Davies NG, Centre for the Mathematical Modelling of Infectious Diseases COVID-19 Working Group, et al. Projecting contact matrices in 177 geographical regions: An update and comparison with empirical data for the COVID-19 era. PLoS Comput Biol. 2021;17: e1009098.
