# Supplementary material for: Estimating the global burden of viable Mycobacterium tuberculosis infection: A mathematical modelling study
Source: PLoS Med. 2026 Feb 5;23(2):e1004920. doi: 10.1371/journal.pmed.1004920 (PMC12893653; doi:10.1371/journal.pmed.1004920)
Supplement: S2 File — GATHER checklist is made available under the Creative Commons Attribution 4.0 (CC BY 4.0) license. The original checklist can be accessed at: https://doi.org/10.1371/journal.pmed.1002056. (PDF) [file pmed.1004920.s002.pdf]

GATHER: Guidelines for Accurate and Transparent Health Estimates Reporting

Checklist of information that should be included in new reports of global health estimates

Item#	Checklist item	Reportedon page #
**Objectives and funding**		
1	Define the indicator(s), populations (including age, sex, and geographic entities), and time period(s) for which estimates were made.	Methods, par 5
2	List the funding sources for the work.	Funding statement
**Data Inputs**		
For all data inputs from multiple sources that are synthesized as part of the study:		
3	Describe how the data were identified and how the data were accessed.	Methods, par 1-3
4	Specify the inclusion and exclusion criteria. Identify all ad-hoc exclusions.	Methods, par 1-3
5	Provide information on all included data sources and their main characteristics. For eachdata source used, report reference information or contact name/institution, population represented, data collection method, year(s) of data collection, sex and age range, diagnostic criteria or measurement method, and sample size, as relevant.	Methods, par 1-3; Text A; Text D; Text E; Table A
6	Identify and describe any categories of input data that have potentially important biases (e.g., based on characteristics listed in item 5).	Discussion, par 5-7
For data inputs that contribute to the analysis but were not synthesized as part of the study:		
7	Describe and give sources for any other data inputs.	Methods, par 1-3; Text A; Text D; Text E; Table A
For all data inputs:		
8	Provide all data inputs in a file format from which data can be efficiently extracted (e.g., aspreadsheet rather than a PDF), including all relevant meta-data listed in item 5. For anydata inputs that cannot be shared because of ethical or legal reasons, such as third-party ownership, provide a contact name or the name of the institution that retains the right to the data.	See GitHub repository: https://github.com/aschwalbc/MtbInf
**Data analysis**		
9	Provide a conceptual overview of the data analysis method. A diagram may be helpful.	Fig 1
10	Provide a detailed description of all steps of the analysis, including mathematical formulae. This description should cover, as relevant, data cleaning, data pre-processing,data adjustments and weighting of data sources, and mathematical or statistical model(s).	Methods, par 1-5; Text G; Text H
11	Describe how candidate models were evaluated and how the final model(s) were selected.	Methods, par 3
12	Provide the results of an evaluation of model performance, if done, as well as the results of any relevant sensitivity analysis.	Fig 2; Table 1; Table 2; Table G; Table H
13	Describe methods for calculating uncertainty of the estimates. State which sources of uncertainty were, and were not, accounted for in the uncertainty analysis.	Methods, par 1-5; Text B; Text E; Text F
14	State how analytic or statistical source code used to generate estimates can be accessed.	See GitHub repository: https://github.com/aschwalbc/MtbInf
**Results and Discussion**		
15	Provide published estimates in a file format from which data can be efficiently extracted.	S1 Data
16	Report a quantitative measure of the uncertainty of the estimates (e.g. uncertainty intervals).	Results; Table 1; Table 2; Table G; Table H; Table M; Table N
17	Interpret results in light of existing evidence. If updating a previous set of estimates, describe the reasons for changes in estimates.	Discussion
18	Discuss limitations of the estimates. Include a discussion of any modelling assumptions or data limitations that affect interpretation of the estimates.	Discussion, par 4

This checklist should be used in conjunction with the GATHER statement and Explanation and Elaboration document, found on gather-statement.org
