# Supplementary material for: Estimating the global burden of viable Mycobacterium tuberculosis infection: A mathematical modelling study
Source: PLoS Med. 2026 Feb 5;23(2):e1004920. doi: 10.1371/journal.pmed.1004920 (PMC12893653; doi:10.1371/journal.pmed.1004920)
Supplement: Attachment — Submitted filename: MtbInf - Review.docx [file pmed.1004920.s005.docx]

**Response to reviewer comments**

We thank the journal and the reviewer for their constructive review of our work. We are also grateful for the extensions provided, which allowed us the time to carefully consider and address the comments in full. We can confirm that no related manuscripts have been submitted or accepted during this time. We have revised the manuscript accordingly. Please find the responses below:

**Editor:**

1. **You are required to complete PLOS’ questionnaire of inclusivity in global research and submit it with your revised paper.**

We have attached PLOS’ questionnaire of inclusivity in global research alongside the rest of the revised files in this submission.

2. **Please upload any figures associated with your paper as individual TIF or EPS files with 300dpi resolution at resubmission.**

We have uploaded all figures in the manuscript as individual TIF files with 300dpi resolution.

3. **At this stage, we ask that you include a short, non-technical Author Summary of your research to make findings accessible to a wide audience that includes both scientists and non-scientists. The Author Summary should immediately follow the Abstract in your revised manuscript. This text is subject to editorial change and should be distinct from the scientific abstract. Ideally each sub-heading should contain 2-3 single sentence, concise bullet points containing the most salient points from your study. In the final bullet point of 'What Do These Findings Mean?', please include the main limitations of the study in non-technical language.**

We have added the Author Summary to the revised manuscript in accordance with the instructions provided.

4. **Please include page numbers and line numbers in the manuscript file. Use continuous line numbers (do not restart the numbering on each page).**

We have added page numbers and continuous line numbers throughout.

5. **Please consider avoiding the use of red and green in order to make your figure more accessible to those with colour blindness.**

Thank you for the suggestion. We have reviewed the colour schemes of the figures using an online tool that simulates how images appear under various types and severities of colour-blindness (<https://www.color-blindness.com/coblis-color-blindness-simulator/>). This prompted us to revise the colours used in Figure 2 of the manuscript to enhance accessibility. The colour schemes of the remaining figures in both the manuscript and supplementary material were found to be consistently clear across different types of colour-blindness and have therefore been retained. While Figure 1 was affected to some extent, its colour scheme is purely decorative and does not carry interpretative value.

**Reviewer #1:**

6. **The authors derive *Mtb* clearance estimates from Horton et al, PNAS 2023. Having reviewed the Horton et al paper, the estimate corresponds to Figure 2. From this, they consider that self-clearance occurs at years 1 (95% CI:80.1-81.7%), 2 (95%CI: 91.4-92.5%), and 10 (95%CI: 96.9-97.5%). There is a little more uncertainty after year 10. I am concerned about the precision of these estimates. E.g. how can we be confident that 80.1- 81.7% of people self-clear by year 1 when this is impossible to measure empirically? For example, could it actually be that *Mtb* viability is often maintained after year 1 but the risk of disease progression declines? The Horton et al study is based on modelling of historic data. I find this level of precision far too narrow to be plausible. As Schwalb et al acknowledge, self-clearance is a primary determinant of their main results. Thus, I think it would be much more realistic to explore a wide range of self-clearance parameters (e.g. 50-90% at year 1… with similar uncertainty at subsequent years).**

Thank you for this thoughtful and important comment. We agree that the uncertainty ranges reported in Horton et al. (*Proc Natl Acad Sci USA*, 2023) are narrow, partly because they are estimates derived from multiple data points. In our original approach, we used these estimates directly as calibration targets, effectively treating them with high precision — comparable to performing a meta-analysis of a meta-analysis. In response to this comment, we have revised our calibration framework to incorporate a wider and more plausible range of uncertainty. Specifically, we derived a median uncertainty on a logit scale from the lower and upper bounds of the proportion self-cleared in the raw data used by Horton et al. We used this median uncertainty to define a logit-normal calibration likelihood, with a precision more representative of the underlying data in Horton et al. We believe these revisions address the concern and strengthen the robustness of our estimates. The updated targets and assumptions are now described in the revised Methods section [lines: 153-156] and supplementary material [SM6, lines: 120-127].

7. **A more detailed summary of how these self-clearance values were estimated, and the underlying assumptions would also be of value in the current manuscript, along with acknowledgement of ongoing uncertainty here as a major (perhaps even critical) limitation.**

Thank you for the suggestion. We agree that, given the central role of self-clearance in this manuscript, it is important to provide more detail on how the calibration targets were derived from the work by Horton et al. (*Proc Natl Acad Sci USA*, 2023). We have added a description of the calculations used to generate these targets in the supplementary material: “*Self-clearance rates were estimated by calibrating to targets based on the proportion of individuals that self-cleared or recovered (i.e., no longer harbouring viable Mtb infection), as described in the natural history of TB model by Horton et al. [10]. In that model, self-clearance from infection was assigned an uninformed uniform prior (U[0.00,6.00] per year), which was constrained through calibration to multiple data: progression following Mtb infection; transitions between non-infectious, asymptomatic, and symptomatic disease states; mortality; duration of infectious TB; and prevalence ratios of non-infectious to infectious TB, and asymptomatic to symptomatic TB.*” [SM6, lines: 114-118].

8. **Following their previous work (Schwalb et al Am J Epidemiol. 2023), the authors adjust their annual risk of infection estimates for reversion. They estimate that the ARI should be inflated x2-5 times to account for this. However, the exact multiplication factor is not presented in the manuscript. From the GitHub code, this appears to be 2.9. I again find this to be a very strong assumption, based on limited data. From reviewing the Am J Epidemiology paper, it seems that underestimation of the ARI was very heterogeneous by age and annual reversion probability (Figure 2 of that paper). Thus, a multiplication factor of 2.9 across the board seems difficult to justify. As for my point above, why not consider a range of estimates (e.g. 2-5x) here to account for the huge amount of uncertainty? I note that in the discussion, the authors allude to age-specific adjustments being unfeasible. This seems like a shame.**

Thank you for this thoughtful comment. We agree that applying a uniform adjustment factor to account for reversion involves important assumptions, and we acknowledge the limitations of this approach. We selected a central value of 2.9 for the reversion adjustment based on our previous work (Schwalb et al., *Am J Epidemiol*, 2023). This value is the median ratio of true to naïve ARI among children aged 8–12 years within the reversion probability range reported by the study by Grzybowski and Allen (*Am Rev Respir Dis, 1964*). Please note that we did incorporate uncertainty around this estimate in our analysis. The uncertainty here is propagated as a factor applied to variances [see *scripts/01_dataprep.R* on GitHub; lines: 66-68, 185, 201, 219], rather than through sampling. In terms of the degree of uncertainty, we applied a 50% increase to the uncertainty of each reversion-adjusted ARI data point. This choice was intended to reflect a broad plausible range around the central estimate, implicitly spanning the adjustment range discussed in the earlier study. Although the 50% scaling was arbitrary, it was adopted as a pragmatic approach to represent substantial uncertainty without introducing a more complex correlation structure, particularly given that uncertainty propagation was already handled through the Gaussian process regression. This rationale, including the adjustment value and relevant uncertainty, has now been described in the supplementary material: “*We used estimates from previous work that quantified how immunoreactivity reversion may lead to downward bias in ARI estimates derived from cross-sectional surveys [11]. Specifically, we applied the median ratio of true to naïve ARI among children aged 8 to 12 years—the most commonly surveyed age group—within the reversion probability range (15.2–31.4%) reported in the study by Grzybowski and Allen, which analysed five consecutive annual TST surveys conducted in Ontario, Canada in 1959 [12]. This yielded an approximate threefold adjustment (2.9) to account for ARI underestimation due to reversion. To account for additional uncertainty introduced by this correction, we increased the uncertainty interval of all reversion-adjusted ARI estimates by 50%.*” [SM5, lines: 96-104].

9. **In the introduction, the authors refer to viable *Mtb* infection as a 'medically actionable target'. But, as discussed later, it isn't really medically actionable if we can't measure it? This speaks to a broader point about the entire analysis - what is the true value of such an analysis and estimation of burden when it is currently not possible to measure? I'd like to see a little more discussion around this, in addition to my points above on unaddressed uncertainty.**

Thank you for this thoughtful comment. We appreciate the concern regarding the measurability of viable *Mtb* infection and its implications for defining it as a medically actionable target. While we agree that viable *Mtb* infection cannot currently be measured directly in clinical or programmatic settings, we consider it medically actionable in the sense that it represents the population eligible for TPT and thus defines an upper bound on who should be targeted. Additionally, these estimates provide important context for evaluating the potential impact of vaccines that aim to reduce progression from viable infection to disease. We have expanded our sentence to clarify what we mean by medically actionable target: “*Focusing on recent viable Mtb infection sets a medically actionable target—serving as a programmatically relevant upper bound—for optimising the prevention cascade and the population to target with TPT [1,3].*” [Introduction, lines: 74-76].

10. **I am surprised that only 1 African country (DR Congo) appears in Table S7 (countries with highest prevalence of viable *Mtb* infection). Can the authors explain this? For example, South Africa has higher annual TB incidence rates than many of the countries listed in the table. Could there be a systematic issue with the analysis approach or underlying assumptions that has led to viable *Mtb* prevalence in African countries being underestimated?**

Thank you for raising this point. This observation has also been noted by colleagues since the preprint was made available. We investigated the representation of African countries in Table S7 (now Table S9), particularly in relation to South Africa. While the HIV adjustment applied to South Africa’s ARI estimates was indeed substantial compared to many countries, the resulting ARI remained relatively high. However, the overall downward trend in ARI over time in South Africa likely contributed to lower recent infection estimates, which may have influenced its omission from the top 10 in the earlier version. During this review, we also identified and corrected two issues. First, the ageing terms were not included in our equations (see response to item 15), and second, the contact mixing matrices used (Prem et al., *PLoS Comput Biol* 2021) have since been updated. As part of our reanalysis—prompted by the widening of self-clearance proportion target ranges—we also incorporated the corrected matrices. As a result of these updates, several African countries now appear in the revised top 10 lists: DR Congo, Nigeria, and South Africa rank among those with the highest number of recent viable *Mtb* infections, while Gabon and the Central African Republic are now included among countries with the highest prevalence. We believe this shift reflects improved alignment of the age structure and contact mixing patterns on the modelled infection burden.

11. **As a more minor point, in the "Regional and country level infection estimates" section of the results the supplementary tables S5, S6 and S7 referenced in the text appear to be different to that in the supplementary material.**

Thank you for pointing this out. We have corrected the references in the manuscript so that they now correspond to the appropriate supplementary tables.

**Reviewer #2:**

12. **Many assumptions (about the relationship between incidence and prevalence, and then the relationship between prevalence and ARI, and also incidence itself is for many countries a model-based estimate) go into the indirect ARI estimates. Since few countries have recent**

**immunoreactivity surveys, it appears that the indirect ARI estimates contribute heavily to the estimates of ARI in recent years in the GP regression. Were the authors able to do any**

**validation of the indirect ARI approach? For example, in country-years that have immunoreactivity surveys, do direct and indirect ARI estimates match?**

Thank you for your question. We agree that the indirect ARI estimates rely on several assumptions, including the relationships between incidence and prevalence, and subsequently between prevalence and ARI. As the reviewer notes, the majority of ARI data points, particularly in recent years, are derived from TB incidence estimates, due to the limited availability of recent immunoreactivity surveys. These incidence estimates are first converted to prevalence, as WHO no longer publishes TB prevalence data. We acknowledge the concern around the reliance on indirect estimates and have sought to incorporate all available data sources to maximise the use of empirical ARI estimates where available. However, formal validation of the indirect ARI approach is not feasible at present due to the limited number of countries and years for which recent immunoreactivity survey data exist. We did explore how prevalence estimates (converted from incidence) compared with TB prevalence survey data in selected settings, but did not perform a systematic comparison between direct and indirect ARI estimates. In addition, while direct ARI estimates remain static, indirect estimates—including those for past years—can change across successive WHO Global TB Report iterations, which further complicates consistent validation. Overall, we view direct and indirect ARI estimates as complementary, with each contributing unique strength. We also agree that formal validation would be valuable but is currently constrained by the limited scope of available empirical data.

13. **On a related note, I was surprised to see the narrowness of the uncertainty intervals presented in tables 1-2, given the large uncertainty intervals in the ARI estimates presented in table S4. I wonder if this may come down too much of the uncertainty getting "averaged out", as a result of how correlations in uncertain parameters (like ARI) are handled across age groups, countries, and years. For example, +/- 50% uncertainty was added to the ARI uncertainty intervals when the 3x adjustment was made. In the 1000 iterations, were higher ARI estimates in 1 country/year/age group paired with higher ARI estimates in other countries/years/age groups, or was each of the 1000 samples from a country-age-year ARI distribution treated as independent from the other country-age-year ARI distributions? It is reasonable to expect that if the 3x adjustment is an overestimate (or underestimate) in 1 setting, age group, or year, it could be an overestimate in other settings, age groups, and years, but also reasonable to expect that some countries would have a different adjustment factor than others (perhaps due to the characteristics of their survey, etc.). Could the authors please clarify how correlations between parameters were handled and consider exploring sensitivity analysis that does include correlations in some parameters (or components of parameter estimation), if correlations are not currently included.**

Thank you for this insightful comment. Another reviewer raised concerns in relation to uncertainty, and we have carried out substantial additional work on this described above in items 6 and 8—particularly in relation to ARI adjustments and self-clearance assumptions. However, we did not include correlations between parameters across settings or model runs. This is now explicitly stated in the manuscript: “*No correlations were assumed between parameters.*” [Methods, line: 172].

14. **Figure 1 is generally very helpful, but some additional explanation (i.e., as a caption) would be appreciated and would make the methods much easier to understand. For example, what do the colours and light vs. dark shading represent? The left-hand side is the most unclear: do arrows between boxes indicate that one box led to another box, or that those two boxes were combined to produce a third box, or both? (For example, did the ARI estimates, country-level TB data, and adjustments for smear positivity all contribute to the revised Stýblo rule, or were they combined with the revised Stýblo rule to produce the combined ARI dataset? Was the Stýblo rule actually applied to the Direct ARI estimates?)**

Thank you for this helpful comment. We have reevaluated Figure 1 and agree that additional explanation in the caption improves its clarity. We have reevaluated Figure 1 and agree that additional detail in the caption improves clarity. The revised caption now better explains the flow between components and clarifies how the various data sources and methods relate to the revised Stýblo rule.

15. **Methods, self-clearance rates and model structure sections: Why are progression and death not modelled as competing risks in either model? Presumably ignoring these competing risks would lead to an overestimation of the prevalence of infection.**

We are very grateful for this question, as it prompted us to check our formulae, during which we realised that the ageing term ($\varepsilon$) had not been included. This has now been corrected (shown in the formulae in SM8 in the supplementary material) and incorporated into our latest model run and results. A notable outcome of this correction can be seen in Figure S6, which now displays a smoother distribution in the proportion of individuals with viable *Mtb* infection across age group. Regarding your question, we did include death as a competing hazard and suspect the confusion may be due to our equations being formulated in terms of proportions rather than population counts. If $X_{\alpha}$ is the susceptible population count, $Y_{\alpha}^{\eta}$ is the infected population count, and the total population in age group $\alpha$ is $N_{\alpha}=X_{\alpha}+\sum_{\eta} Y_{\alpha}^{\eta}$, then our formulation is in terms of the corresponding proportions $S_{\alpha}={X_{a}}/{N_{\alpha}}$ and $I_{\alpha}^{\eta}={Y_{\alpha}^{\eta}}/{N_{\alpha}}$. So, for example, the dynamics for $X$ look like: $X_{\alpha}^{'}=\delta_{1,\alpha}\cdot\theta-\mu_{\alpha}\cdot X_{\alpha}-\lambda_{\alpha}\cdot X_{\alpha}+\sum_{\eta} \gamma_{\eta}\cdot Y_{\alpha}^{\eta}+\varepsilon\cdot[(1-\delta_{1,\alpha})\cdot X_{\alpha-1}-X_{\alpha}]$, which explicitly includes competition with death (the $\mu$ term). Because the total population counts satisfy $N_{\alpha}^{'}=\delta_{1,\alpha}\cdot\theta-\mu_{\alpha}\cdot N_{\alpha}+\varepsilon\cdot[(1-\delta_{1,\alpha})\cdot X_{\alpha-1}-X_{\alpha}]$, the dynamics for $S$ become:

$$S_{\alpha}^{'}=\frac{X_{\alpha}^{'}}{N_{\alpha}}-\frac{N_{\alpha}^{'}}{N_{\alpha}}\cdot\frac{X_{\alpha}}{N_{\alpha}}$$

$$=\frac{1}{N_{\alpha}}\cdot\left( \delta_{1,\alpha}\cdot\theta-\mu_{\alpha}\cdot X_{\alpha}-\lambda_{\alpha}\cdot X_{\alpha}+\sum_{\eta} \gamma_{\eta}\cdot Y_{\alpha}^{\eta}+\varepsilon\cdot\left[ \left( 1-\delta_{1,\alpha} \right)\cdot X_{\alpha-1}-X_{\alpha} \right] \right)$$

$$-\frac{1}{N_{\alpha}}\cdot\left( \delta_{1,\alpha}\cdot\theta-\mu_{\alpha}\cdot N_{\alpha}+\varepsilon\cdot\left[ \left( 1-\delta_{1,\alpha} \right)\cdot N_{\alpha-1}-N_{\alpha} \right] \right)\cdot S_{\alpha}$$

$$=\delta_{\alpha1}\cdot\frac{\theta_{t}}{{fN}_{\alpha,t}}\cdot\left( 1-S_{\alpha} \right)-\lambda_{\alpha,t}\cdot S_{\alpha}+\sum_{\eta=1}^{4} (\gamma_{\eta}\cdot I_{\alpha}^{\eta})+({1-\delta}_{\alpha1})\cdot\varepsilon\cdot\left( S_{\alpha-1}-S_{\alpha} \right)\cdot\frac{{fN}_{\alpha-1,t}}{{fN}_{\alpha,t}}$$

Notice that the mortality terms ($\mu$) cancel out when writing the dynamics in terms of proportions. To avoid confusion for other readers, we have added the following note after the equations in the supplementary material: “*Note that when formulating dynamics in terms of age-stratified proportions in a dynamic population, age-specific mortality rates cancel out.*” [SM8, line: 183-185]. Finally, regarding the inclusion of progression, we assume this refers to progression to disease. In this work, we chose to focus exclusively on *Mtb* infection. Progression to disease is not linear, and the endpoint is not always symptomatic TB, particularly given the dynamic course of TB disease over time. Even if progression had been included in the model, it is unlikely that this would lead to an overestimation of infection.

16. **Methods: 80-82% clearance after 1 year of infection is both very high and very certain. Could the authors please include sensitivity analysis that explores wider variation in self-clearance, or lower self-clearance targets? I don't doubt that self-clearance is higher than most models assume but have trouble believing that we can describe self-clearance over time with this much precision.**

We appreciate that uncertainty around self-clearance emerged as a key concern for both reviewers. Please refer to item 6, where we detail the changes made to this component.

17. **Is there any reason why those who were infected and then self-cleared do not experience protection from reinfection in the model? Presumably some maintain immunoreactivity which could imply immunological memory? If it is uncertain whether this protection factor would apply, perhaps this could be mentioned as a limitation.**

Thank you for this question, as protection from reinfection is indeed an important aspect to account for in TB models. In our model, we opted not to include an explicit compartment for individuals who had cleared infection. Introducing a separate "*Cleared*" compartment—representing uninfected, but previously infected individuals—is indeed how many other models handle self-clearance, and such compartment typically carries partial protection against reinfection. Instead, we accounted for protection from reinfection by adjusting the force of infection experienced by individuals who are already infected, implicitly assuming that prior infection confers reduced susceptibility. This was a modelling choice focused on simplicity and tractability; however, we acknowledge that this assumption may overlook the potential for immunological memory among those who have cleared infection, and we have now noted this as a limitation in the revised manuscript: “*Furthermore, while our model does not explicitly include a compartment for individuals who have self-cleared infection, we account for protection against reinfection through a reduced force of infection for individuals with current infection. However, this approach may underestimate the potential contribution of immunological memory in those who have cleared infection.*” [Discussion, lines: 301-305].

18. **Reversion and self-clearance are almost certainty related phenomena, but they are decoupled in this analysis. Annual reversion of 15-30% from Schwalb et al. 2023 and annual**

**clearance of 80% at year 1 from Horton et al. 2023 do not seem necessarily inconsistent, but a brief discussion of the relationship between reversion and self-clearance would be helpful for contextualizing the analysis.**

Thank you for this comment. We agree that reversion and self-clearance are likely related phenomena (for example, reversion may be observed in individuals who have cleared infection), but in the context of our analysis, they are treated as conceptually distinct. Our considerations around reversion relate specifically to its contribution to the underestimation of ARI, as reversion is not accounted for in its calculation. In contrast, self-clearance refers to the biological process by which viable *Mtb* bacilli are eliminated from the body. We have added a clarification in the manuscript to contextualise this distinction: “*Although reversion and self-clearance are likely related phenomena, they represent conceptually distinct processes in the context of this analysis. Reversion, defined as the loss of immunoreactivity [12], can lead to the underestimation of ARI if not accounted for, as individuals with prior infection may test negative [13,14]. In contrast, self-clearance refers to the biological elimination of viable Mtb bacilli from the body [8,9,16,17]. While reversion may occur in individuals who have self-cleared, not all reversion indicates true clearance, nor does all self-clearance necessarily result in reversion [8].*” [Discussion, lines: 262-268].

19. **Methods, self-clearance rates: it is not clear what the "similar model structure as described above" is referring to. Is it the model structure in the following section?**

Thank you for picking this up. Yes, the sentence was intended to refer to the model structure described in the following section. In a previous draft, the model structure appeared before the self-clearance rates section, and this wording remained unintentionally. We have now revised the sentence in the manuscript to ensure clarity: “*Using a Bayesian approach, self-clearance rates were calibrated by tracking an infected cohort over time, using a simplified version of the model structure described below. This structure comprised sequential infected compartments representing increasing time since infection, allowing for self-clearance while excluding infection or reinfection.*” [Methods, lines: 148-151]. We also amended the text to describe this simplified version of the model in the supplementary material: “*We developed a transition matrix model using a simplified structure similar to the one described below, comprising sequential ‘Infected’ compartments that represent increasing durations since infection and accounting only for self-clearance. The model tracked an infected cohort beginning in the ‘Infected – Year 1’ compartment, with self-clearance and progression through infection-year transitions modelled as competing risks. Infection and reinfection were not included.*” [SM6, lines: 107-111].

20. **Methods, self-clearance rates: can the authors please clarify whether in this step they are calibrating a model to fit estimates of self-clearance that were generated from another model? If this is the case (a) can you please explain why you didn't just use that model's self-clearance rates and (b) where the year 1, 2, and 10 clearance targets are coming from in that paper (Horton et al. 2023)? In the results of that paper, "over a 10-y period following *Mtb* infection, 92.0% (95% UI 91.4 to 92.5) of simulated individuals cleared infection without progressing to TB, with 90.5% (95% UI 89.9 to 91.1) clearing infection within the first 2-y" doesn't match the year 2 and 10 targets reported here.**

Thank you for these detailed questions. Yes, we calibrated self-clearance rates in our model to match the proportions of individuals who had self-cleared or recovered, as estimated by the model presented in Horton et al. (Proc Natl Acad Sci USA, 2023). Regarding the follow-up questions:

a) We did not directly use the self-clearance rates from Horton et al. because their model structure differs substantially from ours. Horton et al. employed an individual-based model that explicitly captures TB progression and regression dynamics, while our model is compartmental, with multiple infected compartments. As such, the self-clearance rates are not directly transferable. Instead, we used their proportion estimates of individuals who no longer harbour viable *Mtb* infection over time as calibration targets.
b) We agree that the quoted results in Horton et al. do not exactly match the proportions we report. The values we used (80.9% at year 1, 91.9% at year 2, and 97.2% at year 10) were provided directly by the lead author (who is also a co-author of this manuscript). These represent the cumulative proportion of individuals in their model who had either self-cleared or recovered—i.e., individuals no longer harbouring viable Mtb infection. This is explicitly described in the Methods section of the manuscript [lines 151-155].
21. **Figure 1: how is the case-detection rate being used? I didn't see this described in the text or appendix but may have missed it.**

Thank you for the comment. The case detection ratio was used specifically to compute the mean TB duration by HIV status for each country-year. This was part of the adjustment applied to account for differences in the proportion of smear-positive TB among people living with HIV when generating the ARI estimates. This methodological step follows the approach described in Houben and Dodd (2014) and is now explicitly clarified in the supplementary material: “*Additionally, ARI estimates were adjusted to account for the proportion of prevalent TB that is smear-positive in children and in people living with HIV, using the case detection ratio to compute the mean TB duration by HIV status for each country-year [2].*” [SM3, lines: 78-80].

22. **Methods: Was the "revised Stýblo rule" re-estimated for the present study (i.e., to include more recent publications and adjust for underestimation of ARI), or was the revised rule from Dodd et al. 2014 used? Could the appendix include a quantitative description of the revised rule?**

Thank you for this question. We confirm that the revised Stýblo rule used in our study is the same as that used in Dodd et al. (*Lancet Glob Health*, 2014) and was not re-estimated for this analysis. The quantitative description of the rule—β ∼ LogNormal(μ = 1.678, σ = 0.371)—has now been added to the supplementary material for clarity: “*As seen in the study by Houben and Dodd [2], a revised Stýblo rule was used by fitting a log-normal distribution to data from more recent ARI and TB prevalence estimates [7]: β ∼ LogNormal(μ = 1.678, σ = 0.371).*” [SM3, line: 77].

23. **Methods: What is the purpose of applying smear-positivity in the construction of the revised Stýblo rule? Are the authors assuming that only smear-positive individuals are infectious?**

Thank you for this question. We do not assume that only smear-positive individuals are infectious. However, the Stýblo rule, in its original and revised formulation, relates specifically to smear-positive TB. In line with previous applications of the revised rule (i.e., in the work by Houben and Dodd in *PLoS Med*, 2014), we applied smear-positivity to the WHO TB incidence estimates to approximate the number of individuals with smear-positive TB, which is the quantity required for use in the Stýblo framework. This step was taken to maintain consistency with the intended application of the rule, rather than to imply that smear-positive cases are the sole contributors to transmission.

24. **Results, Global burden of infection estimates: Were the estimates of recently infected exactly the same across the 2 long-term clearance scenarios? I would expect slight differences due to the protection from reinfection factor (more clearance -> less protection -> more new infections), but maybe they round to the same values? Perhaps this could be clarified in the Table 1 and 2 captions.**

Thank you for this observation. As the reviewer correctly assumed, estimates of recent infection are not identical but differ only minimally. In light of reviewer comments and a new run of the model the estimates of all infections between scenarios are much more similar now. Given this we have opted to report the estimates from the high self-clearance scenario in the main body but provide the results of the low self-clearance scenario in Table S6 and S7 of the supplementary material.

25. **I realise we probably lack the data to inform age- or HIV-status-specific self-clearance rates, but it seems biologically plausible that such variation could exist. Could some discussion of this be added?**

Thank you for raising this. We agree that variation in self-clearance rates by age and HIV status is biologically plausible. However, as noted, we lack data to inform such stratification in our model. We have added a sentence to the discussion to acknowledge this and highlight it as an important area for future research: “*In this context, although self-clearance rates may plausibly vary by age and HIV status, we currently lack the date to inform this heterogeneity. We therefore we did not stratify by these factors in our model, though future work should aim to explore and incorporate such variation where possible.*” [Discussion, lines: 298-301].

Please let us know if we can provide any additional information. We look forward to hearing from you at your earliest convenience.

Warm wishes,

Alvaro Schwalb, for the authors
