# Supplementary material for: Estimating the global burden of viable Mycobacterium tuberculosis infection: A mathematical modelling study
Source: PLoS Med. 2026 Feb 5;23(2):e1004920. doi: 10.1371/journal.pmed.1004920 (PMC12893653; doi:10.1371/journal.pmed.1004920)
Supplement: Attachment — Submitted filename: MtbInf_-_Reviewer_Comments_auresp_4.docx [file pmed.1004920.s007.docx]

**Response to reviewer comments**

We thank the journal and the reviewers for their constructive review of our work. We have revised the manuscript accordingly. Please find the responses below:

**Reviewer #1:**

**Thank you to the authors for their further revisions. The additional sensitivity analyses (Table S13) are very helpful. I have a few more suggestions relating to the self-clearance assumptions and sensitivity analyses.**

Thank you to the reviewer for their helpful comments in the previous round of reviews. We appreciated the detailed feedback, which included sensitivity analyses for self-clearance.

1. **The new finding that "global burden estimates were particularly sensitive to lower self-clearance rates, highlighting the need for better empirical data to inform this parameter" is quite critical and is a key take-home message from this manuscript, since the evidence underpinning the primary self-clearance estimates are so weak. I would strongly suggest that these findings are included in both the abstract and author summary.** We thank the reviewer for raising this point. We agree that it is important to highlight this. We have now included this in both the abstract (“*Sensitivity analyses of varying self-clearance scenarios showed significant changes in global estimates of viable Mtb infection, particularly in total burden, with lower self-clearance rates.*” [Abstract, lines 38-40]), and the author summary (“*Sensitivity analyses reveal that our estimates are sensitive to varying assumptions about self-clearance rates.*” [Author summary, lines 60-61]).
2. **Related, I would also strongly recommend adding a figure that visually represents the findings from table S13 in the main manuscript.** We thank the reviewer for their suggestion. We have added a Figure which visually represents the findings from Table S13 in the main manuscript.
3. **Please can the authors clarify what the self-clearance rate variation of +-25-75% in the sensitivity analyses actually means? For example, how can a baseline assumption of 80.9% self-clearance at 1 year increase by 75% in the sensitivity analysis?** We thank the reviewer for noting this. We agree that it is not so clear as it currently lies. Essentially, we used the median self-clearance rates for the high self-clearance scenario (i.e., Year 1 = 2.13, Year 2 = 1.51, Years 3 to 10 = 0.41, and Years 10+ = 0.07) and adjusted according to the variation (+75%, +50%, +25%, -25%, -50%, -75%). For example, under the +75% increase sensitivity analysis, the values above were multiplied by 1.75, resulting in adjusted medians (Year 1 = 3.73, Year 2 = 2.65, Years 3 to 10 = 0.73, and Years 10+ = 0.12), representing 94.0% self-cleared/recovered at year 1. In contrast, under the -75% increase sensitivity analysis, the values above were multiplied by 0.25, resulting in adjusted medians (Year 1 = 0.53, Year 2 = 0.38, Years 3 to 10 = 0.10, and Years 10+ = 0.02), representing 35.7% self-cleared/recovered at year 1. We have reworded the description of this to make it more explicit that it is a relative variation: “*Second, we conducted a parameter-driven analysis in which self-clearance rates were systematically scaled by ±25%, ±50%, and ±75% relative to the median estimates from the calibrated high self-clearance scenario to explore how these rates influence global burden estimates.*” [Methods, lines 204-207], and “*Estimates reflect systematic variation of self-clearance rates relative to the median estimates from the high self-clearance scenario*.” [Table S13 legend]. Additionally, we have added a column in Table S13 to highlight the proportion self-cleared at year 1 and year 2, which aids in the interpretation of this sensitivity analysis.
4. **The limitations section should explicitly acknowledge the weakness of the underlying data on self-clearance, along with the major impact that this has on the results.** Thank you to the reviewer for this helpful comment. We have further expanded on the limited data on self-clearance and its impact on our estimates: “*Another major limitation concerns the evidence base for self-clearance. Although a few studies summarise supportive evidence [1,4,8,9], empirical data remain limited. As our results show, assumptions about self-clearance are a major source of uncertainty in our estimates. Our sensitivity analysis on systematically varied self-clearance rates showed that global burden estimates were particularly sensitive to lower self-clearance rates, highlighting the need for better empirical data to inform this parameter. Additionally, these self-clearance rates were applied uniformly, but they may plausibly vary by age and HIV status; we currently lack the data to inform this heterogeneity. Future work should explore and incorporate such variation where possible.*” [Discussion, lines 327-334].
5. **The abstract states that "Self-clearance rates were informed by empirical data and modelling estimates". But is this really true, given that only the Horton et al modelling estimates are explained and cited as source data for the self-clearance rates in the methods?** We thank the reviewer for raising this point. This was an oversight. In the abstract, we intended only to allude to the use of self-clearance rates informed by the modelling estimates from Horton et al.: “*Self-clearance rates were informed by modelling estimates*.” [Abstract, lines 29-30]

**Reviewer #2:**

**I appreciate the authors' consideration of my previous comments and would like to thank them for adding ARI estimates to the appendix and for clarifying some points about the methods. I believe the authors are doing their best to be responsive to the reviewers' comments, but I still have some concerns about this analysis. A couple of additional comments for the authors' consideration, based on those responses.** Thank you to the reviewer for their earlier feedback. It prompted clarifications that have strengthened the manuscript. We hope our responses below address the remaining comments.

6. **I share Reviewer 1's apprehension about the handling of uncertainty in this analysis. My specific concerns relate more to the ARI estimation process, including no correlation between the many elements of the ARI estimation process that do not represent meaningful cross-country variation (such as the duration of disease, 2.9x adjustment, and revised Styblo rule) resulting in underestimation of uncertainty at the regional and global level (I was not suggesting that the authors induce 100% correlation between these parameters). I am happy to see the ARI estimates shown in more detail in the appendix, but am concerned about the extent to which direct and indirect estimates do not match (in almost half of the country-years where direct estimates are available, the confidence intervals between direct and indirect do not overlap). While I appreciate the sensitivity analysis on clearance rates, I would like to see some attempt to better address uncertainty in ARI (my suggestion was to induce a correlation between country estimates that could be < 100%, say an arbitrary 50%, but this could be handled differently) or to acknowledge these uncertainties much more in the limitations section.** We appreciate the concerns raised regarding ARI estimation. In response to an earlier comment in the previous revision round, we introduced a sensitivity analysis that correlated self-clearance rates, as we considered this the most biologically justified correlation to evaluate. By contrast, we opted not to do so for the parameters underpinning ARI trajectory estimation. We continue to believe that correlating parameters reflecting between-country differences in disease duration (i.e., the prevalence–incidence ratio) or the revised Stýblo rule adjustments for HIV and age would not be appropriate. Introducing correlation between countries in the ARI trajectories would implicitly assume correlated TB burden estimates that drive transmission. This assumption is not supported by current global burden estimation frameworks (such as WHO and IHME), which treat countries as independent when constructing estimates. As the reviewer noted for the context of our study, applying such a correlation would imply substantially larger uncertainty intervals for global TB burden than those currently reported. As we do not consider TB burden estimates to be correlated across countries, the same logic applies to the ARI, which is derived from prevalence. On another point, even after applying the revised Stýblo rule, discrepancies between these two data sources persist. Both sets of estimates, along with their associated uncertainties, are incorporated into the GP regression, which is designed to accommodate such inconsistencies when projecting ARI trajectories by increasing uncertainty to encompass discrepant data. We have added further details on this and acknowledge these uncertainties explicitly in the Discussion: “*A major limitation concerns the estimation of the ARI. Empirical ARI estimates remain scarce, so contemporary ARIs had to be inferred indirectly from WHO TB incidence estimates, which provided the necessary data points for the GP regression. In country-years where both direct and indirect ARI estimates were available, more than half had non-overlapping uncertainty intervals; however, the GP regression was able to accommodate this discrepancy. Ideally, population-level trends in Mtb transmission should be evaluated through repeated immunoreactivity surveys to obtain direct ARI estimates.*” [Discussion, lines 315-325].
7. **The fact that, in this model, viable infection includes active disease introduces some additional issues that were not apparent previously.** We thank the reviewer for returning to this important point. We appreciate their continuous attention to the fact that our viable infection estimates include TB disease, and agree that it merits a clear and explicit discussion. **The main issue is that, in reality, those with active disease can "clear" their infections (i.e., recover from TB disease) by being treated, not just through natural recovery following disease. In the model, these individuals should therefore face high rates of exit out of "viable infection" - but if I understand correctly this is not being captured because the clearance rates are calibrated to output from Horton et al. 2023, which did not include treatment. Omitting exits due to treatment of active disease is therefore likely to result in an overestimation of the prevalence of viable infection and of recent infection.** The reviewer correctly notes that our model does not capture treatment-driven recovery among individuals with TB disease, as the calibrated clearance rates are based on Horton et al. 2023, which excludes treatment. We agree that this omission is likely to lead to some overestimation of the burden of viable *Mtb* infection. However, this effect is expected to be limited in magnitude: only around 5–10% of those infected ever develop active TB, with most doing so within the first two years of infection. We now state this limitation explicitly in the Discussion: “*Finally, because the calibration targets for the proportion self-cleared or recovered were derived from a model that accounts for progression and regression across multiple TB disease states and does not include treatment-driven recovery, our estimates of viable Mtb infection include individuals with TB disease. As a result, the burden, particularly the fraction that might be considered medically actionable, may represent an upper bound*.” [Discussion, lines 348-353]. **There are some additional concerns that probably have less influence on the overall numbers (although it's difficult to say without any sensitivity analysis on this), and so I will emphasize them less. But they include (1) that people with TB disease should face higher mortality rates. The supplementary materials statement "Note that when formulating dynamics in terms of age-stratified proportions in a dynamic population, age-specific mortality rates cancel out" would seem to no longer apply if mortality rates vary by time since infection, which again they may if the proportion of "clearance" that is due to progression and recovery (vs. infection clearance without ever progressing) is higher during earlier years of infection.** The reviewer notes that individuals with TB disease should experience higher mortality rates, and that our supplementary statement about age-specific mortality cancelling out may not hold if mortality varies by time since infection, given that a greater proportion of TB disease arises from recent infections. We agree that this is plausible, but there are no data to inform the most accurate distribution of TB disease arising from recent versus distal infection. Any unmodelled variation in mortality by time since infection, therefore, forms part of the uncertainty we already acknowledge, which we estimate would affect headline results marginally. **And (2) the model allows for reinfection, with some protective factor, which doesn't really make sense for people with active disease. While it's true that people can be co-infected (or "co-diseased") with multiple Mtb strains, in the model this would send these individuals back to "infection year 1".** We agree that, under our framework, reinfection would reset such individuals to “infection year 1”, which is not biologically appropriate for those with TB disease. However, in our model, reinfection occurs within distal infection, where only a small fraction of TB disease would likely reside; thus, as the reviewer correctly noted, we expect it to have only a minimal effect on the overall degree of overestimation. **Again, I suspect that these two limitations have little influence on the overall numbers generated by this analysis, but the magnitude of overestimation from excluding clearance/recovery due to treatment is less clear, without any sensitivity analyses being run. I agree with the authors that such an analysis would require a more complex model, but perhaps given the limitations of the current approach, a more complex model is warranted.** We agree that a more complex model would allow us to address the points raised above and estimate a viable *Mtb* infection estimate that explicitly excludes individuals with TB disease. However, implementing such an approach would involve not simply adding a disease compartment, but rather several TB disease states. While we recognise the value of such an approach and see it as a natural direction for future work, it is beyond the scope of the present analysis, which is focused on establishing a first global benchmark under a simplified model structure. We have reflected this limitation in the text: “*We recognise that addressing this limitation fully would require a more complex model, incorporating explicit representation of multiple TB disease states and consideration of treatment-driven recovery. We see this as an important and natural direction for future work*” [Discussion, lines 353-356]. **Without any sensitivity analysis on this, my suggestion is that much of the manuscript framing and wording seem to need revision if the estimates of viable infection include people with prevalent/active TB disease.** We have further revised the manuscript to ensure that the framing and wording consistently reflect that our estimates of viable infection include individuals with TB disease. For example, this is now made more explicit in the Methods: “*The model by Horton et al. captures detailed progression and regression across multiple TB states, ranging from Mtb infection through asymptomatic and symptomatic infectious disease. As such, the complement of these calibration targets (i.e., the proportion that does not self-clear or recover), and thus our estimates, represent individuals who harbour viable Mtb bacilli, including those with TB disease*” [Methods, lines 163-168]. **Referring to the rate of transition from infection to uninfected as a "self-clearance" rate seems inaccurate.** We appreciate the nuances raised by the reviewer, but on balance, we feel this is an appropriate term for this context. The transition we are modelling reflects the loss of harbouring viable mycobacteria, and self-clearance remains the dominant process governing this transition in our framework. As such, we don't think that the term warrants a change. We have clarified the definition when first using the term: “*Furthermore, in this study, the term self-clearance is used as an overarching concept encompassing both biological clearance without progression and natural recovery after progression to disease.*” [Methods, lines 168-170]. **As another example, the paper repeatedly alludes to the utility of these estimates in guiding TPT provision, but people who have active TB disease should not be given TPT. As I understand it, we cannot just adjust for the estimates of recent viable infection by subtracting out estimates of active TB prevalence, because we do not know the proportion of active TB prevalence that is from recent infection.** As noted above, our estimates do include individuals with TB disease; however, this represents only a small proportion of the total burden. We fully agree that individuals with active TB disease should not receive TPT. A central aim of our manuscript is to move away from equating immunoreactivity with infection and, instead, to more realistically approximate the population with viable *Mtb* infection, which represents the medically actionable target group for preventive therapy. Regarding the wording of the manuscript: we do not claim that everyone included in our estimates should receive TPT. Rather, we emphasise the need for diagnostic tools capable of identifying those with viable infection, given that tools for identifying disease already exist. **While active disease is likely to represent a minority of infection prevalence, this caveat limits the extent to which the numbers presented are "medically-actionable". I would like to see the framing of the manuscript and some of the model terminology revisited to ensure it is appropriately describing what is actually being estimated.** Overall, we appreciate the point that including TB disease in the estimates will overestimate the burden to an extent. We agree this is useful to reflect on, and thank the reviewer for pushing this point. We have now acknowledged this limitation clearly in both the methods and the interpretation of the results, while also providing a ceiling for the impact.

**Reviewer #3:**

**The authors have revised their paper, and this is now a substantially improved manuscript, acceptable for publication.**

Thank you. We are glad the revisions have improved the manuscript.

Please let us know if we can provide any additional information. We look forward to hearing from you at your earliest convenience.

Warm wishes,

Alvaro Schwalb, for the authors
