# Supplementary material for: Estimating the global burden of viable Mycobacterium tuberculosis infection: A mathematical modelling study
Source: PLoS Med. 2026 Feb 5;23(2):e1004920. doi: 10.1371/journal.pmed.1004920 (PMC12893653; doi:10.1371/journal.pmed.1004920)
Supplement: Attachment — Submitted filename: MtbInf_-_Reviewer_Comments_auresp_5.docx [file pmed.1004920.s008.docx]

**Response to editor requests and reviewer comments**

We thank the editor and the reviewer for their constructive review of our work. We have revised the manuscript accordingly. Please find the responses below:

**Editor:**

1. **Please review your text for claims of novelty or primacy (e.g. 'for the first time') and remove this language. In addition, please check that any use of statistical terms (such as trend or significant) are supported by the data, and if not please remove them.**

We have reviewed the text for claims of novelty or primacy and have removed such language where present. All statistical terms used are supported by the data and have been retained.

2. **In the last sentence of the Abstract Methods and Findings section, please describe the main limitation(s) of the study's methodology.**

Thank you for this comment. We agree with the editor that the main methodological limitation should be clearly indicated in the Abstract Methods and Findings. We have therefore revised the final sentence of this section to explicitly note this: “*Overall estimate uncertainty was considerable, reflecting limitations in the underlying data informing key model parameters.*” In addition, in the Abstract Methods and Findings section, we have further highlighted the limited empirical evidence informing self-clearance: “*Empirical data on self-clearance are limited, so rates were informed by modelling estimates.*”

3. **In the author summary, in the final bullet point of 'What Do These Findings Mean?', please include the main limitations of the study in non-technical language.**

We have followed the reviewer’s suggestion (item #21) for the content of the final bullet point in the Author Summary. We also believe that the wording avoids overtly technical language and clearly conveys the main limitations of the study.

4. **Please add this statement to the manuscript's Competing Interests: "PJD is a paid statistical consultant on PLOS Medicine's statistical board."**

We have edited our statement to include this sentence.

5. **Please convert any stacked bar charts to another data representation, for example, a table, or another type of graph.**

We feel that bar charts remain the most appropriate way to present the data shown in Figs 4 and 5, as they allow readers to directly visualise patterns in recent and distal infection prevalence, with the total height of the bar representing all viable infections, which is of key interest. Presenting these quantities separately using bar charts side by side or any other chart would reduce the clarity with which overall patterns can be assessed. Nonetheless, we agree that the numerical values underlying the figures should be readily accessible in an alternative format. This was already the case for Fig 5, for which values were provided in S14 Table (previously S13 Table) at the request of a reviewer. We have now additionally included the S12 Table, which presents the data shown in Fig 4 for ease of reference.

6. **Please confirm that the appropriate usage rights apply to the use of this map.**

We confirm that vector map data was obtained from Natural Earth, where all maps are in the public domain. In their terms of use (https://www.naturalearthdata.com/about/terms-of-use/), the following statement is there: “*No permission is needed to use Natural Earth. Crediting the authors is unnecessary.*” Despite this, we note the origin of the map data in the legend of Figure 3: “*Vector map data obtained from Natural Earth (https://www.naturalearthdata.com/)*.”

7. **In the final bullet point of ‘What Do These Findings Mean?’ Please include the main limitations of the study in non-technical language.**

As above in item #3.

8. **Please confirm that your title complies with PLOS Medicine's style. Your title must be nondeclarative and not a question. It should begin with main concept if possible. "Effect of" should be used only if causality can be inferred, i.e., for an RCT. Please place the study design ("A randomized controlled trial," "A retrospective study," "A modelling study," etc.) in the subtitle (ie, after a colon). The current title does not adhere to our style.**

We confirm that the title is non-declarative and begins with the main concept. However, to comply fully with PLOS Medicine style, we have revised the title to include the study design too: “*Estimating the global burden of viable Mycobacterium tuberculosis infection: A mathematical modelling study*”.

9. **Please confirm that your abstract complies with our requirements, including format (three sections: Background, Methods and Findings, and Conclusions) and providing all the information relevant to this study type.**

We confirm that our abstract complies with the requirements of PLOS Medicine, including the formatting and the information provided relevant to the study type.

10. **Please ensure that the Introduction ends with a clear description of the study question or hypothesis.**

We confirm that the Introduction ends with a clear description of the study question and the approach used to address it.

11. **Please ensure that all abbreviations are defined at first use throughout the text.**

We have revised the text and ensured that all abbreviations are properly defined at first mention throughout.

12. **Please confirm that all numbers presented in the abstract are present and identical to numbers presented in the main manuscript text.**

We have revised the abstract and confirm that the numbers presented are identical to those in the manuscript text.

13. **The funding statement should include: specific grant numbers, initials of authors who received each award, URLs to sponsors’ websites. Also, please state whether any sponsors or funders (other than the named authors) played any role in study design, data collection and analysis, the decision to publish, or preparation of the manuscript. If they had no role in the research, include this sentence: “The funders had no role in study design, data collection and analysis, decision to publish, or preparation of the manuscript.” The funders' URLs are missing from the statement.**

We have amended the funding statement to include the required sentence: “*The funders had no role in study design, data collection and analysis, decision to publish, or preparation of the manuscript.*” Additionally, we have added the funders’ URLs to the statement.

14. **Please provide a diagram that shows the model structure, including how the disease natural history is represented, the process and determinants of disease acquisition, and how the putative intervention could affect the system.**

The model structure is shown in Fig 1 of the manuscript as part of the conceptual overview of the study, and also as a standalone figure in the supplementary material (S11 Fig). Additionally, the model structure is described in the supplementary material (S7 Text).

15. **Please provide a complete list of model parameters, including clear and precise descriptions of the meaning of each parameter, together with the values or ranges for each, with justification or the primary source cited, and important caveats about the use of these values noted.**

The model parameters are listed in the supplementary material (S3 Table), with descriptions provided for each parameter. Values or ranges are given where applicable, as some parameters are time- and country-dependent. For these, their justification, sources, and caveats are discussed in detail in the manuscript and supplementary texts (S1-S8 Texts) .

16. **Please provide a clear statement about how the model was fitted to the data, including where relevant goodness-of-fit measures, the numerical algorithm used, which parameters varied, constraints imposed on parameter values, and starting conditions.**

Details on the calibration of the annual risk of infection and self-clearance are described in the manuscript, with additional information on self-clearance provided in the supplementary material (S6 Text).

17. **For uncertainty analyses, please state the sources of uncertainties quantified and not quantified. This can include parameter, data, and model structure.**

We recognise the importance of parameter uncertainty and employ methods that explore plausible ranges for key parameters, thereby generating uncertainty intervals for our estimates. Sources of uncertainty that are explicitly quantified include self-clearance rates and the annual risk of infection, with additional uncertainty incorporated for the latter to account for immunoreactivity reversion. Uncertainties that are not fully quantified include the model structure, for example, in representing viable *Mtb* infection in the absence of disease. The details of the uncertainty in parameter values and resulting model outputs are provided in the Methods and Results sections, with further details in the supplementary materials.

18. **Please provide sensitivity analyses to identify which parameter values are most important in the model. Uncertainty estimates seek to derive a range of credible results on the basis of an exploration of the range of reasonable parameter values. The choice of method should be presented and justified.**

Sensitivity analyses are described in the Methods and Results sections of the manuscript, in which we examine the influence of correlation assumptions and of wider variation in self-clearance rates on model outputs, with their implications presented in the Discussion

19. **Please discuss the scientific rationale for this choice of model structure and identify points where this choice could influence conclusions drawn. Please also describe the strength of the scientific basis underlying the key model assumptions.**

The scientific rationale and key assumptions underpinning the model structure are described in the manuscript. We also acknowledge the limitations associated with this choice of model, noting that a more complex structure may be required to fully characterise viable *Mtb* infection in the absence of disease, although this represents a small proportion of our estimates.

**Reviewer #1:**

**Thank you to the authors for the revisions; the manuscript has been further improved. Some minor (final!) suggestions.**

Thank you for the comments in all the review rounds. They have improved the manuscript substantially.

20. **Given the considerable uncertainty, please highlight this in the final sections of the abstract and author summary. E.g: Abstract Conclusions: "Our findings offer the first global burden estimates of viable Mtb infection, albeit with considerable uncertainty owing to weak underlying data."**

Thank you for this comment. We understand the request to emphasise the uncertainty arising from limitations in the underlying data. Rather than explicitly characterising the data as weak, we highlight both the limited empirical evidence informing self-clearance and the resulting uncertainty in the estimated burden of viable *Mtb* infection in the Abstract Methods and Findings (see response to editor’s request #2). This addresses the concern regarding uncertainty without qualifying the Abstract Conclusions, which we consider should focus on the implications of the findings rather than restating methodological caveats.

21. **Author Summary What do these findings mean?: "Future estimates would benefit from more contemporary, empirically derived annual risk of infection data through repeated immunoreactivity surveys, along with empirical measures of self-clearance."**

Thank you for noting this. We agree that future estimates would also benefit from empirical measures of self-clearance, and the text has been revised accordingly.

Please let us know if we can provide any additional information. We look forward to hearing from you at your earliest convenience.

Warm wishes,

Alvaro Schwalb, for the authors
