# Supplementary material for: Estimating the global burden of viable Mycobacterium tuberculosis infection: A mathematical modelling study
Source: PLoS Med. 2026 Feb 5;23(2):e1004920. doi: 10.1371/journal.pmed.1004920 (PMC12893653; doi:10.1371/journal.pmed.1004920)
Supplement: Attachment — Submitted filename: MtbInf - Reviewer Comments.docx [file pmed.1004920.s006.docx]

**Response to reviewer comments**

We thank the journal and the reviewer for their constructive review of our work. We have revised the manuscript accordingly. Please find the responses below:

**Reviewer #1:**

**Many thanks to the authors for the time and effort taken to address the previous comments. They have clearly sought to do a thorough job. I do have some remaining concerns/comments relating to the robustness of the results.**

Thank you for your helpful comments. We really appreciated the detailed feedback in the first round, as it helped us improve the manuscript considerably.

1. **I am still quite nervous about the precision of the estimates provided. Notably, they have changed considerably in the revised version, compared to the original. For example, 289 (95%UI:242-343) million people, representing 3.7% (95%UI: 3.1-4.3) of the global population, are now estimated to harbour a viable Mtb infection. This was previously stated in R1 as between 387 (95%UI:347-441) and 606 (95%UI:549-670) million people, or between 4.9% (95%UI:4.4-5.6) and 7.7% (95%UI:6.9-8.5) of the global population. Notably, the 95% uncertainty intervals between the original and new estimates do not overlap. I appreciate that the authors have re-run their analyses, including aiming to account for more uncertainty in clearance of infection estimates and correcting for issues with ageing terms and contact mixing matrices. But it is quite disconcerting that the main analysis findings can change beyond the widths of the presented uncertainty intervals, between revisions. To me, this perhaps reflects the underlying truth that there is a great deal of uncertainty that underlies the present analysis. My strong preference would be to embrace and tackle this uncertainty head-on (see comment 3 below), rather than risking "burying it" deep in the methods.**

Thank you for this comment. We appreciate the concern regarding the change in estimates between manuscript versions. However, it is important to note that uncertainty intervals reflect statistical uncertainty around model inputs and outputs—they do not reflect bias. As such, revised point estimates may fall outside previous intervals when removing an inadvertent source of bias (i.e., changing ageing and mixing) without implying that the uncertainty intervals are too narrow. Thanks to your comments and those from other reviewers, we introduced several key improvements to the model. Specifically, we corrected the application of ageing terms in the ODE equations (we apologise for this error, and thank the reviewer for helping spot it), updated the contact mixing matrices, and expanded the uncertainty bounds used for calibrating self-clearance targets. These changes contributed to shifts in the total infection estimates but also led to broader uncertainty intervals. For example, the relative width of the 95%UI for the total infection estimates increased from 20-24% to 35%, better reflecting uncertainty in key inputs and assumptions. We would also like to emphasise that estimates of recent infection remained very stable across revisions (134 million [95% UI: 104–171] vs. 156 million [95% UI: 127–199]), suggesting that the most policy-relevant estimate of the target population for preventive treatment has remained robust.

2. **Related, the authors state that "Estimates of both overall infection burden and recent infection were robust across scenarios (Table S6 and Table S7)." However, Tables S6 and S7 do not seem to present any alternative scenarios, unless I have missed these? They seem to show numbers and proportions of individuals with viable Mtb infection by region. If this is the case, why are the results now robust, when they previously varied depending on long-term self-clearance assumptions?**

Thank you for raising this point. In the original manuscript, we referred to the robustness of recent infection estimates, which showed minimal variation across the two self-clearance scenarios. In the revision, we reported these explicitly, and they remained highly consistent (134 million [95%UI: 104-171] in the high self-clearance vs 134 million [95%UI: 104-174] in the low self-clearance scenario). Following the revised analysis, which included broader uncertainty bounds for the self-clearance targets, we also observed that estimates of total infection were now more similar and with overlapping uncertainty intervals across scenarios (289 million [95%UI: 242-343] in the high self-clearance vs 322 million [95%UI: 271-389] in the low self-clearance scenario). Accordingly, we updated the revised manuscript to reflect that both recent and total estimates were robust, highlighting the stability of these results across scenarios. We have revised the language in the manuscript to clarify this: “*Estimates of both total and recent infection were robust across scenarios, with minimal differences observed between the high and low long-term self-clearance assumptions.*” [Results, lines 226-227]. Furthermore, the intended comparison is between Table 1 and Table S7 [previously S6] (number of individuals with viable *Mtb* infection) and between Table 2 and Table S8 [previously S7] (proportion of the population with viable *Mtb* infection). We have revised the manuscript text to clarify this comparison: *“(Table 1 vs Table S7 for absolute numbers; Table 2 vs Table S8 for proportions of the population)”* [Results, lines 227-228].

3. **I appreciate that the authors have now tried to incorporate uncertainty in the self-clearance estimates (shown in Figure S4). As a non-mathematical modeller, I am unclear on how to interpret the approach and on how it influences the downstream analyses. Do the final modelled estimates of viable Mtb infection include the uncertainty bounds shown in the self-clearance calibration plot (Figure S4), or do only the point estimates really get pulled through? I would find this much easier to interpret if the authors could provide current Mtb viability estimates where the self-clearance rates are systematically varied across a plausible range (I previously suggested 50-90% or similar at 1 year, with a trajectory thereafter that reflects this). Otherwise, the findings risk being fundamentally based on calibration to a previous natural history mathematical model (Horton et al.) that was itself based on historic data, but without any supporting empirical data. This feels quite risky given that the final publication is likely to be highly cited. I believe that my suggestion to clearly show the impact of systematically varying the self-clearance assumption (for which we have very weak underlying data) on the final estimates will be much more meaningful and interpretable to the broad readership of PLOS Medicine.**

Thank you for this helpful comment. We appreciate your suggestion to make the impact of self-clearance more interpretable to readers. We would like to highlight two key points. First, the final modelled estimates of viable *Mtb* infection do incorporate the full range of uncertainty reflected in the self-clearance calibration plot (Figure S10, previously S4). Second, while the data informing self-clearance targets are indeed limited, we reiterate that these analyses represent the most robust approach we can take given the currently available evidence. That said, we conducted a sensitivity analysis that is parameter-driven and deliberately divorced from the empirical data that was or could be used to inform the self-clearance targets (e.g., Behr et al. *Br Med J* 2019 review; calibration to progression by Horton et al. *Proc Natl Acad Sci* 2023; autopsy studies reviewed by Emery et al. *Proc Biol Sci* 2021). This analysis explores how the global burden estimates vary when self-clearance rates are systematically scaled by ±25%, ±50%, and ±75% from the high self-clearance scenario. We have added details of this analysis: “*Second, we conducted a parameter-driven analysis in which self-clearance rates were systematically scaled by ±25%, ±50%, and ±75% from the point estimates of the calibrated high self-clearance scenario, to explore how these rates influence global burden estimates.*” [Methods, lines 198-200]; and report the results in the revised manuscript: “*Under varying self-clearance scenarios, global estimates of viable Mtb infection increased with lower self-clearance rates, particularly for total burden (Table S13). The proportion of infections that were recent declined as total burden rose accordingly. At higher self-clearance levels, total infection estimates remained relatively stable.*” [Results, lines 256-259]. Additionally, we added some reflection of this in the Discussion: “*Our sensitivity analyses—examining both correlated self-clearance parameters across countries and systematically varied rates—showed that global burden estimates were particularly sensitive to lower self-clearance rates, highlighting the need for better empirical data to inform this parameter.*” [Discussion, lines 329-332]. Please also see our response to comment 4, where we include an additional analysis that compares results under correlated versus uncorrelated self-clearance assumptions across countries. Both helps illustrate how self-clearance affects our estimates and uncertainty.

**Reviewer #2:**

**I would like to thank the authors for their revisions to their paper (particularly the revised approach to estimating clearance rates that results in increased uncertainty) and thoughtful responses. I have a couple of remaining concerns about the revised manuscript that should be possible to address through further revisions.**

Thank you for your previous comments and for acknowledging the revisions we made. We appreciate the constructive points raised and aim to address them below.

4. **Regarding the previous comment #13 in the response document, I appreciate the authors clarifying that no correlations were included in parameters across countries, years, or age groups. However, I am still concerned that the lack of correlations, particularly in ARI estimates across countries, results in the uncertainty in country-specific values being to a large extent "washed out" in regional and global estimates - because the analysis is much more likely to pair high ARI samples from some countries with low samples from others, rather than including high samples from all countries - when in reality it is possible that components of the ARI estimation process (such as the 2.9x adjustment factor) result in systematic over- or under-estimation. For example, comparing Table S9 to Table 1, we can see that there is much more uncertainty at the country level than at the regional level. I would request again that the authors consider at least a sensitivity analysis that induces correlations across countries, or some other method that makes it more likely to sample high (or low) ARI values across most countries in the same run. If I understand the methods correctly, I don't think this would require rerunning the analysis - just combining results across countries differently.**

Thank you for this comment. While we appreciate the rationale for considering complete correlation of parameters across countries in ARI estimates, we do not believe this assumption is appropriate. These estimates reflect true between-country differences in transmission dynamics, influenced by varying epidemiological and demographic factors. Therefore, we consider it fitting to treat these as uncorrelated. However, we agree that for parameters representing biological processes, such as self-clearance, it is more plausible that values may be similar across settings. To explore this, we conducted a sensitivity analysis in which a defined set of self-clearance parameters was drawn and applied uniformly across countries, rather than independently sampling values for each country and model run. As with our other sensitivity analysis on varying self-clearance rates, this analysis illustrates how assumptions about self-clearance affect the uncertainty of our results. We have added a description of this sensitivity analysis: “*First, we assumed that self-clearance parameters were correlated across countries: for each model run, a sampled set of self-clearance values was drawn and applied uniformly to all countries, reflecting the assumption that natural history processes may be consistent across settings.*” [Methods, lines 195-198]; and present the corresponding results in the revised manuscript: “*Under the scenario assuming correlated self-clearance rates across countries, the estimated burden of viable Mtb infection in 2022 was similar to the main analysis, though with wider UIs: 287.2 million people (95%UI: 196.1–393.8) in total, and 132.2 million people (95%UI: 79.5–202.2) with recent infection (Table S12).*” [Results, lines 253-256].

5. **I understand that a formal validation procedure for comparing indirect and direct ARI estimates presents a number of challenges. Could the authors at least include an appendix table that compares direct ARI estimates (e.g., perhaps those from the past 20 or so years from Table S1) with the indirect ARI estimate for a corresponding age-country-year? I understand that a unique strength of the indirect estimates is that they are readily available for all countries and years, but if they have another advantage over the direct estimates, that is expected to result in differences between the direct and indirect estimates that would be helpful to explain.**

Thank you for this helpful suggestion. We agree that, while a formal validation is not feasible, a side-by-side comparison between direct and indirect ARI estimates can offer useful insights. We have now added a supplementary table comparing these two estimates (prior to reversion adjustment and GP regression) in country-years where both are available and noted this in the revised manuscript: “*For country-years in which both direct and indirect ARI estimates were available, these are compared in Table S2.*” [Methods, lines 127-128]. Additionally, we include GP regression runs for all countries in the supplementary material to illustrate how these inputs shape the final ARI trajectories: “*For each country, we generated 1,000 simulated ARI trajectories spanning 1950 to 2022 (Figures S1–6).*” [Methods, lines 133-134].

6. **Regarding the previous comment #15, I agree that progression is non-linear and disease states after progression can be dynamic, but couldn't an exit from viable Mtb infection resulting from progression be modelled that matches the estimates from Horton et al. 2023? In that paper, by year 1 and beyond, the prevalence of "active" TB exceeded that of "viable infection" - so it would seem that after an initially high clearance rate, the dynamics of disease progression would start to become an important input into the number of viable infections that remain. Furthermore, it appears that progression is included in the model used to calibrate the clearance rates, so using those calibrated rates in a model without progression is somewhat problematic. The statement "Even if progression had been included in the model, it is unlikely that this would lead to an overestimation of infection" may be true, but it seems very speculative at this point, and I am not convinced (but am open to convincing, as I may have missed something). If not, perhaps a sensitivity analysis that includes some exit due to progression based on the Horton et al. paper could be a good solution to determine if this is indeed the case. If that is not possible, I'd like to see some attempt by the authors to address this in some other way.**

Thank you for this detailed and considered comment. You have highlighted an important point that we had not made sufficiently explicit. Our modelling approach defines viable *Mtb* infection to include individuals with TB disease, as these individuals necessarily harbour viable bacilli. This is consistent with the ICE-TB classification of early TB states (Coussens et al., *Lancet Respir Med* 2024). As such, exits from the infection pool due to progression to disease are not modelled as removals, because disease represents a continuation of viable infection rather than a resolution of it. Our calibration to self-clearance rates, therefore, captures individuals who no longer harbour viable *Mtb*, whether through immunological clearance or natural recovery following disease. We acknowledge this was not sufficiently clear previously and have now added text in the revised manuscript to clarify this assumption: “*As we calibrated to the proportion who self-clear or recover, our estimates include individuals with TB disease, since they necessarily harbour viable bacilli.*” [Methods, lines 161-163]. Furthermore, we have revised wording throughout the manuscript to avoid implying a stricter separation between viable *Mtb* infection and TB disease, which we hope makes this clearer, and addresses the insightful and valuable comment: “*Additionally, improved characterisation of viable Mtb infection—as part of the ongoing pathological process within the spectrum of TB disease—is needed to better identify and target individuals in whom typical bacteriological confirmation is not possible.*” [Introduction, lines 105-107]; “*These findings underscore the need for enhanced diagnostic and management strategies to identify individuals with viable Mtb infection—particularly those who may benefit from TPT.*” [Discussion, lines 266-268]; among others. Additionally, we comment on the implications of this assumption as a limitation: “*Moreover, as a result of our modelling assumptions, individuals with TB disease were included in our estimates of viable Mtb infection; this means that the total burden may represent an upper bound, particularly for those with distal infection.*” [Discussion, lines 336-338]. Adding an analysis with an exit from infection to disease and/or treatment and cure (thereby restricting the definition of viable *Mtb* infection to exclude pathology) would require a substantial additional model development to properly represent the natural history of TB and stay in line with the other empirical data underlying the parameterisation. We agree that this is an important area for future work and hope to explore it further in subsequent studies.

Please let us know if we can provide any additional information. We look forward to hearing from you at your earliest convenience.

Warm wishes,

Alvaro Schwalb, for the authors
